# Adult porcine genome-wide DNA methylation patterns support pigs as a biomedical model

**DOI:** 10.1186/s12864-015-1938-x

**Published:** 2015-10-05

**Authors:** Kyle M. Schachtschneider, Ole Madsen, Chankyu Park, Laurie A. Rund, Martien A. M. Groenen, Lawrence B. Schook

**Affiliations:** Department of Animal Sciences, University of Illinois, Urbana, IL USA; Animal Breeding and Genomics Center, Wageningen University, Wageningen, The Netherlands; Department of Animal Biotechnology, Konkuk University, Gwangjin-gu, Seoul South Korea; Institute for Genomic Biology, University of Illinois, Urbana, IL USA; 1201 W Gregory Drive #382 ERML, Urbana, IL 61801 USA

**Keywords:** DNA methylation, Pigs, RNA-seq, Biomedical research, Adaptive evolution

## Abstract

**Background:**

Pigs (*Sus scrofa*) provide relevant biomedical models to dissect complex diseases due to their anatomical, genetic, and physiological similarities with humans. Aberrant DNA methylation has been linked to many of these diseases and is associated with gene expression; however, the functional similarities and differences between porcine and human DNA methylation patterns are largely unknown.

**Methods:**

DNA and RNA was isolated from eight tissue samples (fat, heart, kidney, liver, lung, lymph node, muscle, and spleen) from the adult female Duroc utilized for the pig genome sequencing project. Reduced representation bisulfite sequencing (RRBS) and RNA-seq were performed on an Illumina HiSeq2000. RRBS reads were aligned using BSseeker2, and only sites with a minimum depth of 10 reads were used for methylation analysis. RNA-seq reads were aligned using Tophat, and expression analysis was performed using Cufflinks. In addition, SNP calling was performed using GATK for targeted control and whole genome sequencing reads for CpG site validation and allelic expression analysis, respectively.

**Results:**

Analysis on the influence of DNA variation in methylation calling revealed a reduced effectiveness of WGS datasets in covering CpG rich regions, as well as the usefulness of a targeted control library for SNP detection. Analysis of over 500,000 CpG sites demonstrated genome wide methylation patterns similar to those observed in humans, including reduced methylation within CpG islands and at transcription start sites (TSS), X chromosome inactivation, and anticorrelation of TSS CpG methylation with gene expression. In addition, a positive correlation between TSS CpG density and expression, and a negative correlation between TSS TpG density and expression were demonstrated. Low but non-random non-CpG methylation (<1%) was also detected in all non-neuronal somatic tissues, with differences in tissue clustering observed based on CpG and non-CpG methylation patterns. Finally, allele specific expression analysis revealed enrichment of genes involved in metabolic and regulatory processes.

**Discussion:**

These results provide transcriptional and DNA methylation datasets for the biomedical community that are directly relatable to current genomic resources. In addition, the correlation between TSS CpG density and expression suggests increased mutation rates at CpG sites play a significant role in adaptive evolution by reducing CpG density at TSS over time, resulting in higher methylation levels in these regions and more permanent changes to lower gene expression. This is proposed to occur predominantly through deamination of 5-methylcytosine to thymidine, resulting in the replacement of CpG with TpG sites in these regions, as indicated by the increased TSS TpG density observed in non-expressed genes, resulting in a negative correlation between expression and TSS TpG density.

**Conclusions:**

This study provides baseline methylation and gene transcription profiles for a healthy adult pig, reports similar patterns to those observed in humans, and supports future porcine studies related to human disease and development. Additionally, the observed reduced CpG and increased TpG density at TSS of lowly expressed genes suggests DNA methylation plays a significant role in adaptive evolution through more permanent changes to lower gene expression.

**Electronic supplementary material:**

The online version of this article (doi:10.1186/s12864-015-1938-x) contains supplementary material, which is available to authorized users.

## Background

Epigenetic mechanisms are stable and heritable modifications responsible for regulating gene transcription without altering the DNA’s nucleotide sequence [1]. DNA methylation is a well understood epigenetic mechanism [2]. Vertebrate DNA methylation involves the addition of a methyl group to cytosine bases predominantly located 5’ adjacent to a guanine (CpG sites) throughout the genome. DNA methylation is associated with gene expression and can be altered during responses to environmental factors [1], representing a link between gene expression and the environment. This relationship provides a potentially important role for DNA methylation in adaptive evolution, as it is believed that regulatory changes in gene expression play a particularly important role in the evolutionary process [3, 4]. Additionally, the risk of developing diseases such as diabetes, cardiovascular disease, and cancer has been linked to environmental insults early in life and throughout adulthood. Therefore it should be no surprise that aberrant DNA methylation patterns have been detected in the tissues of individuals suffering from many human diseases including neurodegenerative disorders, diabetes, and cardiovascular disease [5], as well as colorectal [6], ovarian [7], prostate [8], lung [9], and breast cancers [10]. Such studies highlight the importance of methylation patterns and their potential use as biomarkers for early detection, diagnosis and prognosis of complex, chronic diseases.

Recently pigs have emerged as an essential relevant biomedical model due to their anatomical, genetic and physiological similarities with humans, as well as their broad availability, short generation interval and large litter sizes [11–13]. Pigs provide ideal animal models, as porcine studies have been shown to be in general more predictive of therapeutic treatments in humans than rodents [11], and pigs are also less expensive and ethically more acceptable than canines and non-human primates. There is a vast amount of research on the genetic and environmental interactions associated with complex polygenic physiological traits in pigs, making them relevant models for obesity, female health, cardiovascular disease, nutritional and communicable disease studies [13]. Additionally, pigs represent excellent models for defining how environmental signals such as food, smoking, alcohol, and stress affect DNA methylation and contribute to chronic diseases [14]. Currently there are a number of available porcine models of human diseases which have been associated with environmental insults and/or aberrant DNA methylation patterns including cardiovascular disease [15–17], diabetes mellitus [18, 19], Alzheimer’s disease [20], obesity [21], necrotizing enterocolitis [22], and cancer [23].

The recent completion of the swine reference genome sequence [24] further enhances the ability of researchers to perform porcine genotype-phenotype studies for many human diseases, as well as opens the door for targeted approaches to produce tailor-made disease models [25]. Several projects, including the Human ENCODE Project [26], the Human Epigenome Project [27], the International Human Epigenome Consortium [28], and the NIH Roadmap Epigenomics Program [29] have begun to profile epigenetic patterns in a variety of human cell types and conditions, with the goal of producing maps of epigenetic patterns and provide insights into their role in normal and diseased states. While multiple methylation studies have been performed in pigs to date, the majority of these are limited to a small number of tissues and techniques with either limited resolution or limited genomic coverage [30–34]. As the connection between DNA methylation and human disease continues to be developed, there is a growing need to increase the availability of porcine epigenetic resources to fully utilize pigs as human disease models, as well as understand the importance of epigenetic patterns in the process of evolution.

This study produced DNA methylome maps and gene transcription profiles of eight tissues from the adult Duroc female used for production of the swine reference genome [24], reporting baseline porcine DNA methylation patterns similar to those observed in human somatic cells, including a negative correlation between transcription start site (TSS) CpG methylation and gene expression, and a positive correlation between gene body CpG methylation and gene expression. These results support the increased study of DNA methylation patterns and their associations with the development, detection, and potential treatment of relevant human diseases in a porcine model. The additional positive correlation observed between TSS CpG density and gene expression, shown to result from the high rate of 5-methylcytosine (CpG) deamination to thymidine (TpG), provides evidence for a role of DNA methylation in adaptive evolution through more permanent changes to lower gene expression.

## Methods

### Ethics Statement

Tissue collection was conducted at slaughter according to relevant national and international guidelines. Tissue collection procedures were approved by The University of Illinois Institutional Animal Care and Use Committee (IACUC; Protocol numbers 99252 and 04006).

### Sample Collection

Tissue samples (fat, heart, kidney, liver, lung, lymph node, muscle, and spleen) were collected from the adult female Duroc preferentially used for sequencing of the swine reference genome (Duroc 12-4) [24]. The female Duroc was maintained in a purebred breeding herd monitored for clinical disease and management through an active vaccination program, strategic medication and medicated feeds. The vaccination program consisted of vaccinations for *Mycoplasma hyopneumoniae*, *Pasturella multocida* type D, *Erysipelothrix rhusiopathiae*, Parvovirus, leptospirosis, *E. coli*, and *Clostridium perfringens* type C. All animals were inspected daily for health status, and never displayed any symptoms of diseases nor were treated. Although animals sequenced to generate reference genomes tend to be inbred, and it is not uncommon for purebred livestock herds to have an inbreeding coefficient of around 3% due to population structure, the pedigree for this individual indicates an inbreeding coefficient of 0%. Therefore, biases in the analysis due to regions of homozygosity are not expected. The female Duroc was 3 years 4 months of age at the time of euthanasia, at which point tissue samples were harvested and stored at -80^o^C until processing.

### DNA and RNA Isolation

Genomic DNA (gDNA) and total RNA were extracted simultaneously from frozen tissue samples using the AllPrep DNA/RNA Mini Kit (Qiagen, Valencia, CA, USA) following the manufacturer’s protocol. The DNA concentration was determined using a NanoDrop spectrophotometer and DNA quality was assessed by electrophoresis using a 1% agarose gel. RNA concentrations were determined using a NanoDrop spectrophotometer and analyzed by an Agilent 2100 Bioanalyzer using an RNA Nano bioanalyzer chip to determine RNA integrity as well as the presence/absence of gDNA by the Carver High-Throughput DNA Sequencing and Genotyping Unit (HTS lab, University of Illinois, Urbana, IL, USA). Only RNA samples with a RNA integrity number greater than 7 were used for sequencing.

### RRBS and RNA-seq Library Preparation

High-quality gDNA (2 μg) was used for generation of reduced representation bisulfite sequencing (RRBS) and targeted control libraries by the HTS lab (University of Illinois, Urbana, IL, USA) following standard protocols. Briefly, gDNA was restriction digested using the methyl-insensitive restriction enzyme Mspl, which cuts the DNA at CCGG sites. The fragments were blunt-ended and phosphorylated, and a single A nucleotide was added to the 3' ends of the fragments in preparation for ligation to a methylated adapter with a single-base T overhang. The ligation products were purified and size-selected (30 - 160 bp) using agarose gel electrophoresis. Size-selected DNA was bisulfite-treated with the EpiTech Bisulfite Kit (Qiagen, Valencia, CA, USA) and column-purified. The treated DNA was PCR-amplified to enrich for fragments with adapters on both ends. The final libraries were quantified using Qubit (Life Technologies, Carlsbad, CA, USA) and the average size was determined on an Agilent bioanalyzer DNA7500 DNA chip (Agilent Technologies, Wilmington, DE, USA) and diluted to 10 nM. The 10 nM dilution was further quantitated by qPCR on an ABI 1900 to ensure high accuracy quantification for consistent pooling of barcoded libraries and maximization of the number of clusters in the Illumina flowcell. Targeted control libraries were produced in the same manner as RRBS libraries for each tissue type, with the exception of the bisulfite treatment step, in order to validate CpG sites.

High-quality RNA (1 μg) was used to generate TruSeq Stranded RNA-seq libraries (TruSeq Stranded RNA Sample Preparation Kit, Illumina, San Diego, CA, USA) by the HTS lab (University of Illinois, Urbana, IL, USA) following standard protocols. Briefly, messenger RNA was isolated from the high quality DNAse treated total RNA and first-strand synthesis performed with a random hexamer and SuperScript II (Life Technologies, Carlsbad, CA, USA). Second-strand synthesis was performed using dUTP instead of dTTP. Double stranded cDNA was blunt-ended, 3’-end A-tailed and ligated to indexed adaptors. The adaptor–ligated double-stranded cDNA was amplified by PCR for 10 cycles with the Kapa HiFi polymerase (Kapa Biosystems, Woburn, MA, USA) to reduce the likeliness of multiple identical reads due to preferential amplification. The final libraries were quantified prior to cluster generation as described above.

### Illumina Sequencing

RRBS and RNA-seq Illumina sequencing was performed on libraries multiplexed and loaded onto 8-lane flowcells for cluster formation and sequenced on an Illumina HiSeq2000. The libraries were sequenced to a total read length of 100 bp from one end (RRBS and targeted control libraries, single-end sequencing) or both ends (RNA-seq, paired-end sequencing) of the molecules. The run generated .bcl files which were converted into demultiplexed compressed fastq files using Casava 1.8.2 (Illumina, San Diego, CA, USA).

### RRBS Data Analysis

An average of 50.3 million raw reads were produced for each sample, ranging from 36 to 72.3 million (Additional file 1: Table S1). The average bisulfite conversion rate was 94.27 %, ranging between 93.48 % and 94.90 %. Raw reads were trimmed for adapter contamination, minimum quality score (20), experimentally introduced cytosines, and minimum length (20bp) using Trim Galore v.0.3.3 [35]. An in silico converted reduced representation swine genome was produced using the BS-seeker2 v.2.0.5 [36] bs_seeker2-build.py script with fragment lengths between 20 and 180 bp (-r –l 20 –u 180). Trimmed reads were aligned to the in silico converted reduced representation swine genome with BS-seeker2 v.2.0.5 using Bowtie2 v.2.1.0 [37] in local alignment mode, with a seed length of 20, a maximum of 1 mismatch allowed in the seed alignment, and allowing no more than 2 mismatches/read. Methylation level (defined as the ratio of methylated/total reads at a given site) was determined using the bs_seeker2-call_methylation.py script using only uniquely aligned reads. CpG sites from each strand were combined, and all analyses were performed using sites covered by a minimum of 10 reads in all samples to avoid biases caused by varying sequencing depth.

All statistics were performed by importing the data into R v.3.1.2 [38]. Cluster analysis was performed using pvclust v.1.3.2 using the ward.D2 method with 10,000 bootstraps [39]. Methylation levels of individual CpG sites within a given region were used for statistical comparisons within tissues, while the average level for a given region in each tissue was used when comparing across tissue types. For statistical comparisons, TSS regions were defined as 300 bp upstream to 200 bp downstream of the annotated TSS. Gene body regions were defined as the annotated gene region excluding the 5’ 5 % of the gene body, in order to avoid the TSS region. CpG, TpG, and ApG densities were compared across genomic regions by calculating the number of sites within a given set of regions and normalizing to a length of 100 bp. Normality of data was tested using the Shapiro-Wilk or Kolmogorov-Smirnov normality test when appropriate, and an F-test was used to test for equality of variance between groups. Significant differences between groups were determined using the Student’s t-test for normally distributed data, and the Wilcoxon signed-rank test for non-normally distributed data. Spearman’s rank correlation analysis was performed for all correlation analysis. Gene regions with a minimum of 2 high confidence CpG sites were used when correlating CpG methylation with CpG density and/or expression, with the exception of CpG islands (CGIs), which were required to contain a minimum of 4 high confidence CpG sites due to their higher coverage in the RRBS datasets.

### WGS, Targeted Control Data Analysis, and SNP Validation

Whole genome sequencing (WGS) Illumina reads from the same individual were downloaded from the European Nucleotide Archive [ENA:PRJEB9115] and aligned against the swine reference for SNP discovery. Reads were trimmed with Trim Galore v.0.1.4 to a Phred quality > 20 and minimum length of both pairs of 25 bp, and the quality trimmed reads were aligned to the swine reference genome using the unique alignment option of Mosaik Aligner v.1.1.0017 [40]. PCR duplicates were removed using Picard version 1.99 [41] and re-aligned with GATK version 2.3-9-ge5ebf34 [42]. The final alignment of uniquely aligned paired-end reads had an average coverage of 25X with 2.04 GB of the genome covered. Additionally, the targeted control datasets for each tissue were combined, resulting in a total of 136.6 million raw reads. Raw reads were trimmed as described above. Trimmed reads were aligned to the swine reference genome with bowtie2 v.2.2.3 in --end-to-end mode and setting the –N option to 1 and the –L option to 20. The resulting alignments were filtered for uniquely aligned reads, and realigned using GATK v.2.3-9-ge5ebf34. The final alignment of uniquely aligned reads had an average coverage of 12.7X with 0.15 Gb of the genome covered. The resulting targeted control and WGS alignments were used for variant calling using GATK v.3.3-0 with the --stand_call_conf option set to 50, the --stand_emit_conf option set to 20, and the -dcov option set to 200. Variants were filtered so that only SNPs with a read depth between 1/3 and 1.75 times the average coverage (4 – 20 reads for targeted control, 8 – 44 reads for WGS) and a minimum mapping quality of 20 were used for analysis. Depth cutoffs were based on previous studies using similar restrictions [43–45]. Due to the difference in average depth between the WGS and targeted control datasets, a ratio of the average coverage was used to determine the minimum depth cutoff, making sure the minimum number of reads was ≥ 4. Additionally, while many studies use a maximum depth of 2x the average coverage, a conservative cutoff of 1.75x was used in order to reduce the number of false positives detected due to the higher coverage. These sites were removed from the RRBS dataset before analysis.

### RNA-Seq Data Analysis

An average of 45.8 million raw stranded paired-end reads were produced for each sample, ranging from 39.6 to 52.5 million (Additional file 1: Table S1). Raw reads were trimmed sequentially for adapter contamination, A-tails, and minimum quality score (20) and minimum length (20 bp) as described above. Unpaired reads were retained with a minimum length of 35 bp. Trimmed paired and unpaired reads were aligned to the swine reference genome using Tophat v.2.2.10 [46]. Tophat analysis included a pre-alignment to the reference genome to filter out reads extending the maximum number of alignments (-M option) followed by alignment to the Ensembl swine reference transcriptome (-G) and alignment to the genome. The number of allowed alignment hits (-g option) was 20 for differential expression analysis and one for allelic expression (unique alignments only). Furthermore the --read-realign-edit-dist option was set to 0, the --mate-inner-dist option to 120, the --mate-std-dev option to 260 and included the fr-firststrand option. Aligned bam files were assessed for differential gene expression using cufflinks v.2.2.1 [47]. First cufflinks was used to assemble transcripts for each sample using the fr-firststrand option, followed by Cuffmerge to merge the assembled transcripts from all samples with the reference transcripts. Cuffquant was used to pre-compute gene expression levels for each sample using the –u option, which more accurately weights reads mapping to multiple locations, and the fr-firststrand option. Finally, Cuffnorm was used to produce gene expression levels normalized for library size by setting the --library-norm-method to geometric and including the fr-firststrand option.

### Allelic Expression Analysis

Allelic expression analysis was performed following previously published protocols [48]. The RNA-seq alignments were re-aligned for allelic expression with GATK v2.3-9 and PCR duplicates were removed using Picard v.1.119. WGS data from the same individual [ENA:PRJEB9115] was aligned, PCR duplicates removed, and realigned as described above, and variants were filtered so that only heterozygous SNPs with a read depth between 8 (1/3 the average coverage) and 44 (1.75 times the average coverage) and a minimum mapping quality of 20 were used for analysis. The resulting alignment was used for variant calling using GATK v.3.3-0 with the --stand_call_conf option set to 50, the --stand_emit_conf option set to 20, and the –dcov option set to 200. Variation calling in the RNA-seq alignment was performed using SAMtools mpileup v.0.1.18 [49] with a minimum of 10 reads/site and a minimum quality score of 20. The samase.pl script [48] was used to parse the mpileup file, map each variant to an annotated gene, and conduct a binomial test for allele specific expression (ASE) with the --calculate_ase_normal option, and the –r option set to 0.5. ASE positions were removed if they were found to be within 20 bp of another SNP, within 15 bp of an INDEL, had a low calling indication or ratio of <0.3 between SNP reads. Only genes in which all SNPs were found to be ASE SNPs (Benjamini-Hochberg corrected q < 0.05) were used for analysis. GO term and KEGG pathway enrichment analysis was performed for genes displaying ASE in at least 2 tissues using DAVID v6.7 [50, 51] in order to limit any tissue specific bias, with only significantly enriched GO terms and KEGG pathways reported (Benjamini-Hochberg corrected q < 0.05). The background for GO term and KEGG pathway enrichment analysis was all genes tested for ASE (genes containing SNPs).

### Independent validation of gene expression and DNA methylation patterns

In order to validate the results presented in this study, DNA was extracted from 3 tissues (liver, muscle, and spleen) collected from an unrelated adult male Duroc (3 years old) using the DNeasy tissue kit (Qiagen, Venlo, NL, USA). The purified DNA was sent to BGI (China) for RRBS library production as described above. The RRBS libraries were produced using 40 – 220 bp and 220 – 400 bp fragments and sequenced to varying depths using both paired- and single-end sequencing (Additional file 2: Table S2). Raw reads were trimmed, aligned, and methylation levels determined as described above. In addition, overlaps between paired-end reads were removed using the BamUtil v.0.5.7 clipOverlap function after alignment and before methylation calling [52], in order to avoid counting overlapping reads twice. Validations were performed by determining the 10 CpG sites showing the largest increase and decrease in methylation level for each tissue comparison from the adult female Duroc (liver vs. muscle, liver vs. spleen, and spleen vs. muscle) and comparing them to the differences observed at the same sites (60) in the validation dataset. Sites returning a value of –infinity or infinity for log2 fold change (a change from the umethylated (0 %) to methylated state between tissues) were given a value of -6 and 6 for correlation analysis, respectively. This value was chosen in order to give these sites the largest fold change difference of the sites tested.

Total RNA was isolated with the RNeasy mini kit (Qiagen, Benlo, NL, USA). The purified RNA was sent to the HTS lab (University of Illinois, Urbana, IL, USA) for TrueSeq Stranded RNA-seq library production as described above. The RNA-seq libraries were sequenced to an average depth of 35.2 million paired-end reads (Additional file 2: Table S2). The raw reads were trimmed, aligned, and expression levels determined as described above. Validations were performed by calculating the log2 fold change from the 10 most up- and downregulated genes between each tissue comparison from the adult female Duroc and comparing them to the differences observed for the same genes (60) in the validation dataset.

## Results

### Whole genome sequencing and targeted control libraries for CpG site validation

A total of 1,140,814 CpG sites were covered by at least 10 reads in one of the tissue samples analyzed (high confidence sites). These sites were validated using a WGS dataset from the same individual to test for SNPs, since CpG dinucleotides mutate at a high rate due to the deamination of 5-methylcytosine to thymidine [53] and such SNPs could potentially bias the methylation results. Surprisingly, only 334,598 (29.33 %) of the high confidence sites met the depth requirements for SNP detection (see Methods), with 5,515 sites (1.65 %) found to contain SNPs. In order to increase the coverage of high confidence sites for CpG site validation, a control dataset was produced in the same manner as the RRBS datasets, without bisulfite treatment (targeted control). A total of 988,885 (86.68 %) of the high confidence sites met the depth requirements for SNP detection in the targeted control dataset and were tested for CpG site validation. Of these sites, 6,585 (0.67 %) were found to contain SNPs.

In order to investigate the low coverage of CpG sites in the WGS dataset, read depth analysis was performed on both the targeted control and WGS datasets. While a normal distribution was observed for the WGS dataset when considering all genome positions (Additional file 3: Figure S1a), the coverage of high confidence CpG sites revealed a skewed pattern, with the majority of sites covered at levels below the threshold for SNP calling (Additional file 3: Figure S1b). The opposite pattern was observed in the targeted control dataset, with, as expected from a reduced library, a skewed distribution over the whole genome and a more even distribution over the high confidence CpG sites (Additional file 3: Figure S1c, d). When assessing high confidence CpG sites covered in both datasets, 5,017 CpG sites were found to contain SNPs in one of the datasets, of which 1,872 (37.31 %) were detected in both datasets. In total, 10,227 CpG sites were found to contain SNPs in the two datasets combined, and were excluded from further analysis.

### Genomic location of CpG sites targeted by RRBS

Of the CpG sites remaining after SNP removal, a total of 529,902 sites were covered in all samples and used for comparative analyses (Additional file 4: Table S3). While RRBS has been used extensively in human and mouse studies [26, 54], the use of this technology has been limited in pigs to date [22]. Therefore, attention was focused on the distribution of the covered CpG sites in relation to genomic features of interest. Of the CpG sites covered in all samples, the majority were associated with CGIs and CpG island shores (CGS; 64 %; Additional file 5: Figure S2a) or gene bodies (including up to 10 kb upstream of TSS; 66 %; Additional file 5: Figure S2b). In addition, 45.59 % of the CpG sites associated with CGIs were also associated with genic regions, and vice versa (Additional file 5: Figure S2c). The frequency of methylated CpG sites formed a bimodal distribution typical of previously reported RRBS studies (Additional file 5: Figure S2d) [55]. As expected, the number of covered CpG and non-CpG sites on each chromosome was positively correlated with the number of chromosomal CpG sites (Spearman’s Rho > 0.85, *p* < 0.00001; Additional file 5: Figure S2e, f), indicating a lack of chromosomal bias.

### Genome-wide CpG methylation and density patterns in relation to genomic features

**Fig. 1:**
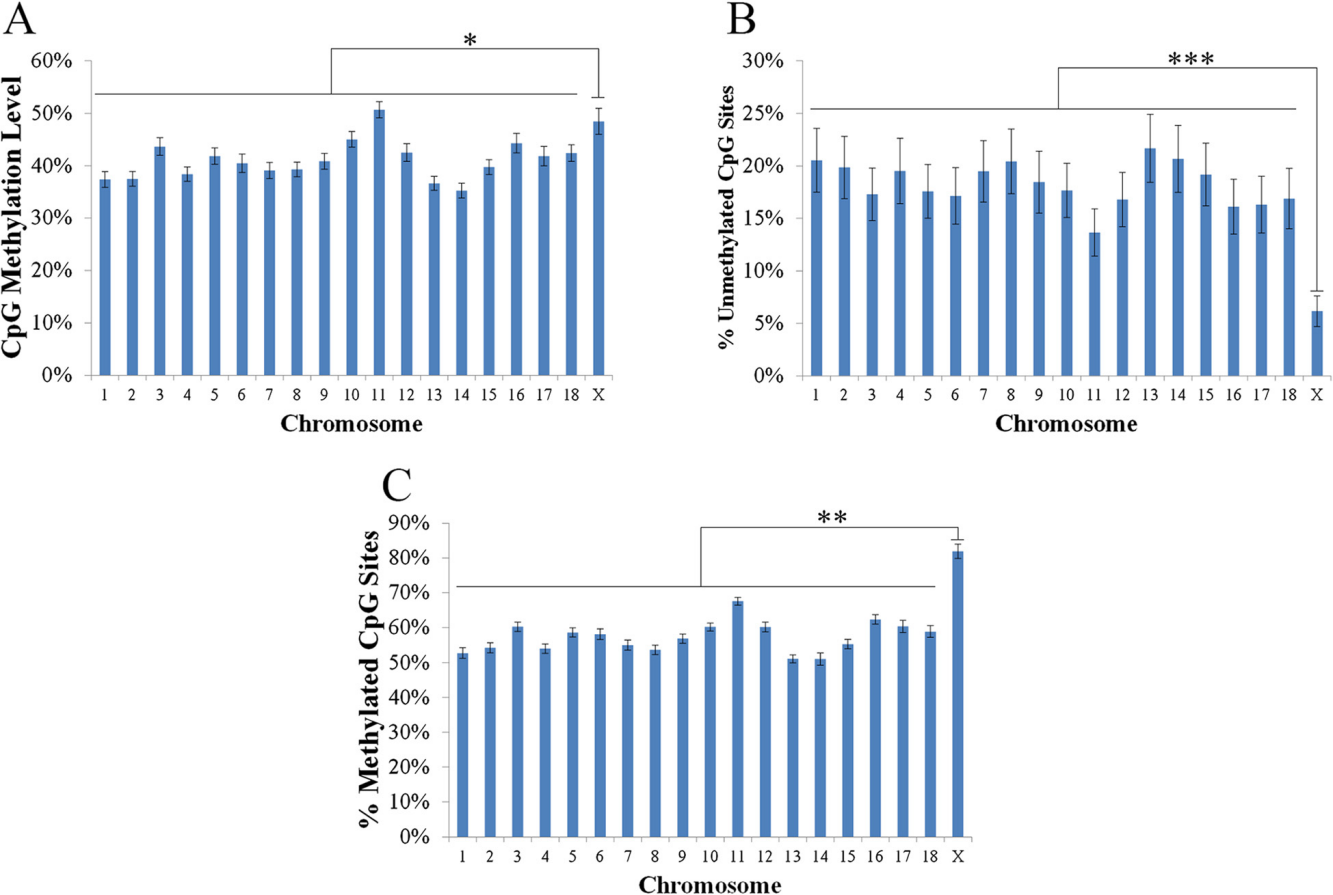
Distribution of CpG methylation across chromosomes. **a** Average CpG methylation levels of all 18 autosomes and the X chromosome across all tissues. Average distribution of **b** unmethylated (0 %) and **c** methylated (>10 %) CpG sites across all tissues for all 18 autosomes and the X chromosome. * denotes *p*-value < 0.0001, ** denotes *p*-value < 1×10^−5^, *** denotes *p*-value < 1×10^−9^

CpG methylation levels (defined as the ratio of methylated reads/total reads at a given site) were similar between tissues, with an average methylation level of 41.39 % ranging between 39.52 % in muscle and 44.75 % in spleen (Additional file 4: Table S3). On average 18.02 % of CpG sites were unmethylated (0 %) in each tissue, ranging from 13.09 % in spleen to 21.5 % in lung, and 57.73 % of CpG sites were methylated (>10 %) in each tissue, ranging from 56.17 % in lymph node to 60.9 % in spleen (Additional file 6: Table S4). When comparing CpG methylation levels across chromosomes, a significantly higher average methylation level was observed for the X chromosome (48.46 %) than the autosomes (40.94 %) across all tissues (*p* < 0.0001; Fig. 1a, Additional file 7: Table S5). This translated to a significantly lower percentage of unmethylated (0 %) CpG sites on the X chromosome compared to the autosomes (6.16 and 18.28 %, respectively, *p* < 1×10^–9^; Fig. 1b, Additional file 8: Table S6), and a significantly higher percentage of methylated (>10 %) CpG sites (81.93 and 57.28 %, respectively, *p* < 1×10^–5^; Fig. 1c, Additional file 9: Table S7). The increased CpG methylation level, reduced proportion of unmethylated CpG sites, and increased proportion of methylated CpG sites on the X chromosome were not observed in the male samples used for validation (see Methods; Additional file 10: Table S8), suggesting that the observed differences in relation to autosomes in the female samples is a result of X chromosome inactivation. Surprisingly, chromosome 11 was found to have the highest CpG methylation level of all the chromosomes in each tissues analyzed (Fig. 1a, Additional file 7: Table S5), however, this was not associated with the same decrease in unmethylated and increase in methylated CpG sites as observed on the X chromosome (Fig. 1b, c, Additional file 8: Table S6, Additional file 9: Table S7).

**Fig. 2:**
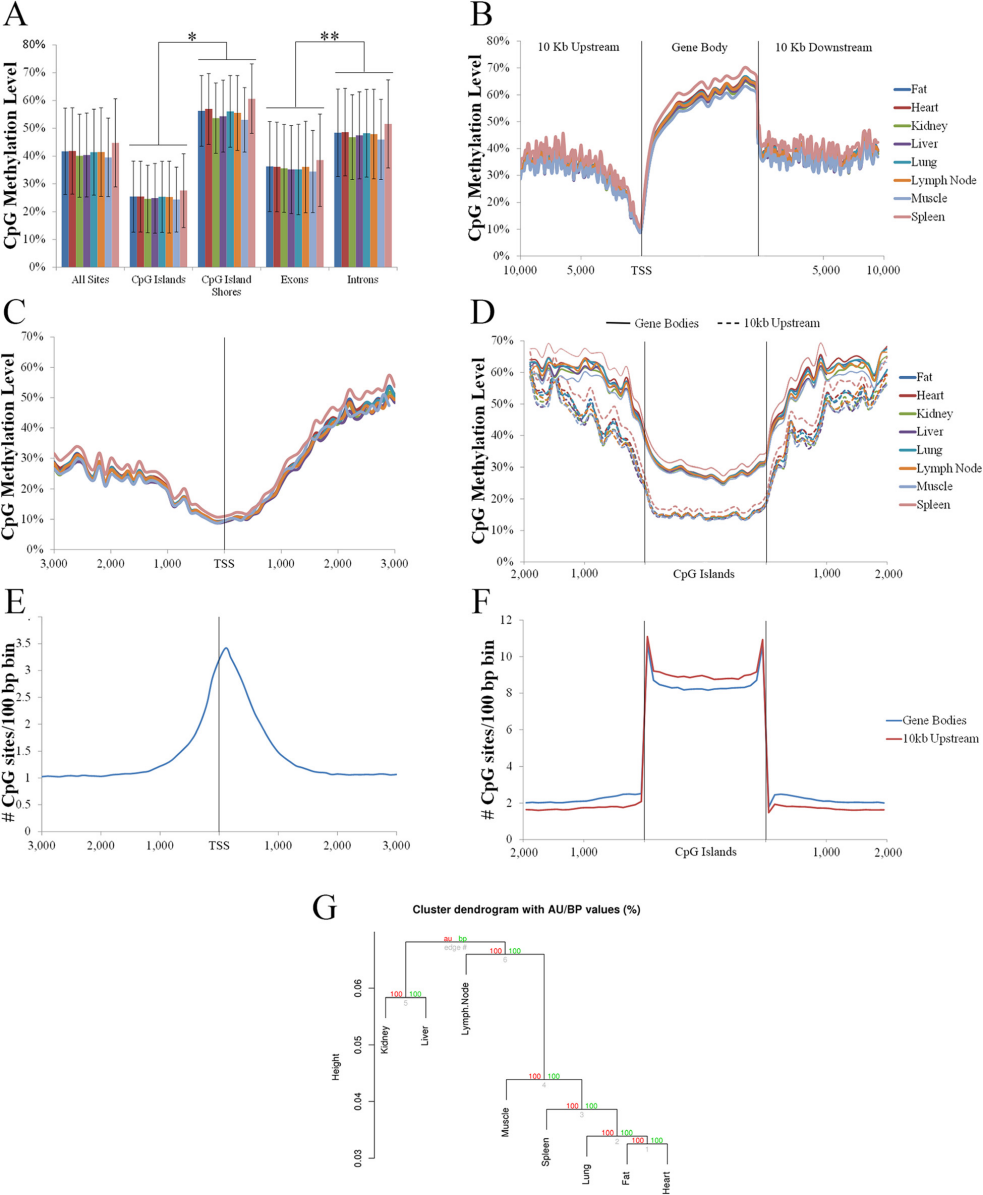
CpG methylation in relation to genomic features across tissue types. **a** Average CpG methylation levels for various genomic features. CpG methylation levels in relation to (**b**) genic regions, (**c**) TSS, (**d**) CGI and CGS within gene bodies and up to 10 kb upstream of TSS. Average CpG density/100 bp bin in relation to (**e**) TSS and (**f**) CGI and CGS within gene bodies at 10 kb upstream of TSS. X axis represents distances in bp. **g** Cluster analysis based on methylation levels at all shared CpG sites, with red numbers indicating the approximately unbiased p-value, and green numbers indicating the bootstrap probability value. * denotes *p*-value < 0.001, ** denotes *p*-value < 1×10^−10^

Profiling CpG methylation patterns within genomic regions revealed similar patterns across all tissue types (Fig. 2a, Additional file 4: Table S3). As expected, significantly lower CpG methylation levels were observed within CGIs compared to CGS both across and within all tissue samples (*p* < 0.001; Fig. 2a, Additional file 4: Table S3). Consistent with previous human studies [56], CGIs and CGS 10 kb upstream of TSS were hypomethylated compared to CGIs and CGS within gene bodies (*p* < 0.001; Fig. 2d). In addition, significantly higher levels of methylation were observed in introns compared to exons (*p* < 1×10^−9^; Fig. 2a, Additional file 4: Table S3). Each tissue also displayed a similar dip in methylation centered at the TSS (Fig. 2c) that increased towards the 3’ end of gene bodies (Fig. 2b). Despite the similarities observed in global CpG methylation patterns, tissue specific methylation patterns were still apparent (Fig. 2g). Surprisingly, tissues with similar functions, such as muscle (skeletal muscle and heart) and immune tissues (lymph node and spleen) did not cluster together.

As CpG methylation is known to be negatively correlated with CpG density [57], the number of CpG sites in the pig genome were profiled in relation to TSS and CGIs, regions in which reduced CpG methylation was detected in all tissue types. As expected from the definition of CGIs, CGIs on average had a higher density of CpG sites than CGS, highlighted by a peak in CpG density at the CGI CGS boundaries (Fig. 2f). Additionally, a higher CpG density was detected within CGIs located within 10 kb upstream of TSS compared to those within gene bodies, with the opposite pattern being observed for CGS (*p* < 1×10^−15^; Fig. 2f). Surprisingly, while a negative correlation between CpG density and methylation was observed in CGIs located within 10 kb upstream of TSS (Spearman’s rho −0.30 to−0.33, *p* < 1×10^−15^) and within gene bodies for all tissue types (Spearman’s rho −0.52 to−0.54, *p* < 1×10^−15^; Fig. 2d, f), a positive correlation between CpG density and methylation was observed in CGS within 10 kb upstream of TSS (Spearman’s rho 0.22 to 0.25, *p* < 1×10^−15^) and within gene bodies for all tissue types (Spearman’s rho 0.21 to 0.24, *p* < 1×10^−15^; Fig. 2d, f). The same correlations were observed when comparing all CGI (Spearman’s rho −0.52 to−0.54, *p* < 1×10^−15^) and CGS in the pig genome (Spearman’s rho 0.22 to 0.25, *p* < 1×10^−15^). Increased CpG density was also observed in relation to TSS, with a negative correlation between CpG density and TSS CpG methylation observed in all tissue types (Spearman’s rho −0.28 to−0.31, *p* < 1×10^−15^; Fig. 2c, e), as is consistent with previous human studies [58].

### Non-CpG methylation distributed in a non-random pattern across tissue types

**Fig. 3:**
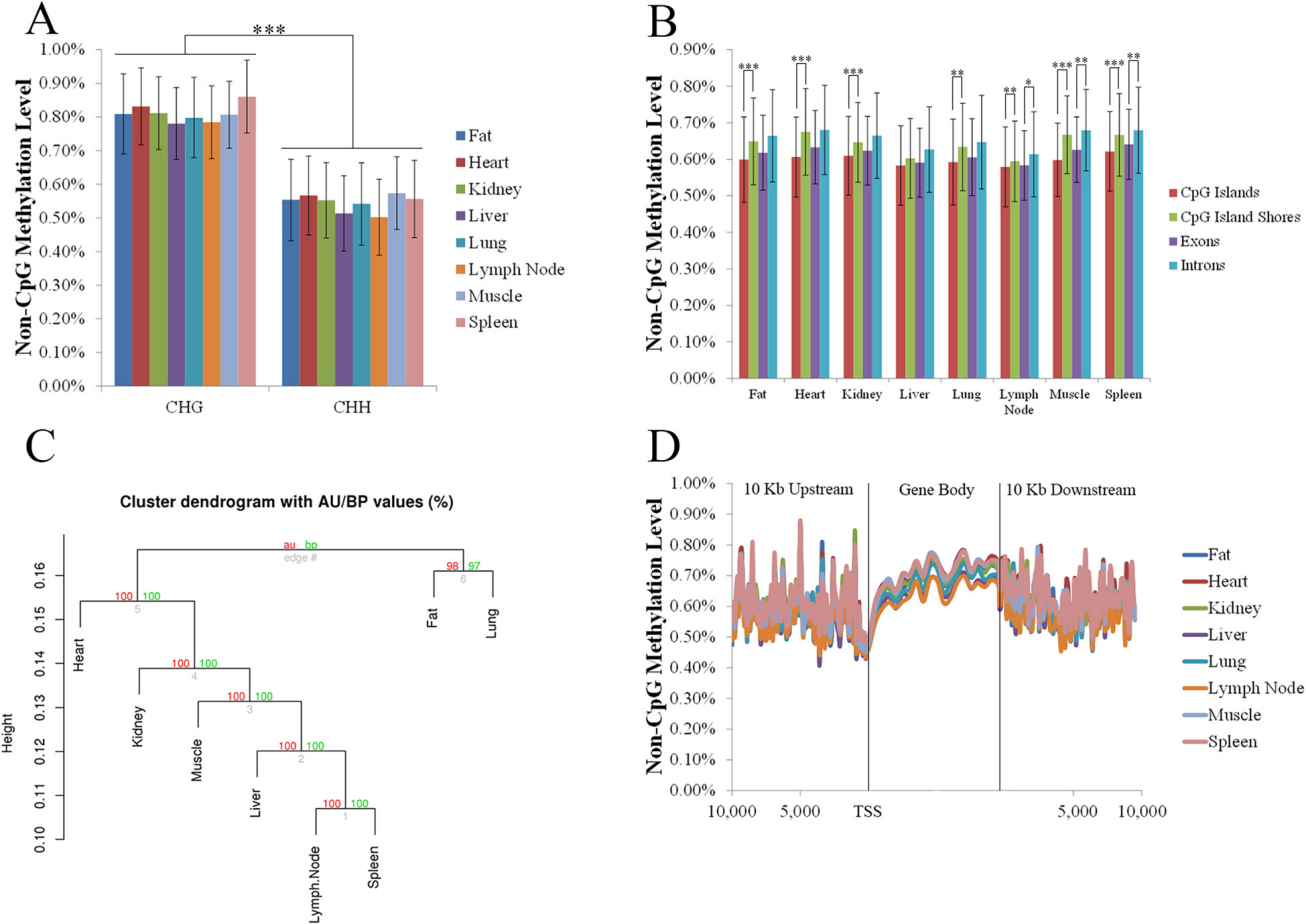
Non-CpG methylation across genomic features. Average methylation level of (**a**) CHG and CHH sites, and (**b**) non-CpG sites in relation to genomic features. **c** Cluster analysis based on methylation levels at all shared non-CpG sites, with red numbers indicating the approximately unbiased *p*-value, and green numbers indicating the bootstrap probability value. **d** Non-CpG methylation levels in relation to gene bodies and TSS. X axis represents distances in bp. * denotes *p*-value < 0.01, ** denotes *p*-value < 0.001, *** denotes *p*-value < 1×10^−10^

In addition to CpG sites, a total of 5,029,035 non-CpG sites were covered by at least 10 reads in at least one sample (high confidence sites). Non-CpG sites covered by either the targeted control or WGS datasets (44.18 % of high confidence sites) were evaluated for SNPs, including the presence of a guanine in the 3’ adjacent base that would result in the presence of a CpG site. Of these, 4,123 (0.19 %) were shown to contain SNPs and excluded from further analysis. A total of 2,191,233 non-CpG sites were covered in all samples after SNP removal and used for comparative analysis (Additional file 11: Table S9). Of the covered non-CpG sites, 63.77 % were CHH sites (H representing nucleotides C, T, or A), with the remainder being CHG sites. The non-CpG methylation levels were similar across all tissue types (<1 %) and consistent with what has been previously reported in porcine non-neuronal tissue (Additional file 11: Table S9) [22]. Additionally, significantly higher levels of methylation were observed at CHG compared to CHH sites when comparing across all tissues (*p* < 1×10^−11^; Fig. 3a) and within tissues (*p* < 1×10^−16^; Additional file 12: Table S10).

No differences in terms of non-CpG methylation were observed between the X chromosome and the autosomes, with an average of 79.01 % of non-CpG sites unmethylated (0 %) and 17.79 % methylated (>1 %) across all tissue types (Additional file 13: Table S11, Additional file 14: Table S12), suggesting non-CpG methylation is not involved in X chromosome inactivation in adult somatic tissues. When comparing non-CpG methylation across genomic regions, a pattern similar to that for CpG sites within CGIs, CGS, introns, and exons was detected, albeit at a much lower methylation level (Fig. 3b). As with CpG sites, significantly lower non-CpG methylation was observed within CGI compared to CGS when comparing across all tissues (*p* < 0.01) and within all tissues except liver (*p* < 0.001; Fig. 3b). In addition, significantly higher levels of non-CpG methylation were observed in introns compared to exons when comparing across all tissues (*p* < 0.01). When comparing within tissue types, significant differences were observed in lymph node, muscle, and spleen (*p* < 0.01; Fig. 3b). Cluster analysis based on non-CpG methylation showed a different pattern than CpG methylation, with immune tissues (lymph node and spleen) clustering together, and fat and lung clustering separately from all other tissues (Fig. 3c). In addition, when non-CpG methylation was profiled in relation to gene bodies, lower methylation at the 5’ end of genes that reached its lowest point at the TSS was observed (Fig. 3d).

### Gene expression, DNA methylation, and CpG density provide potential link to adaptive evolution

**Fig. 4:**
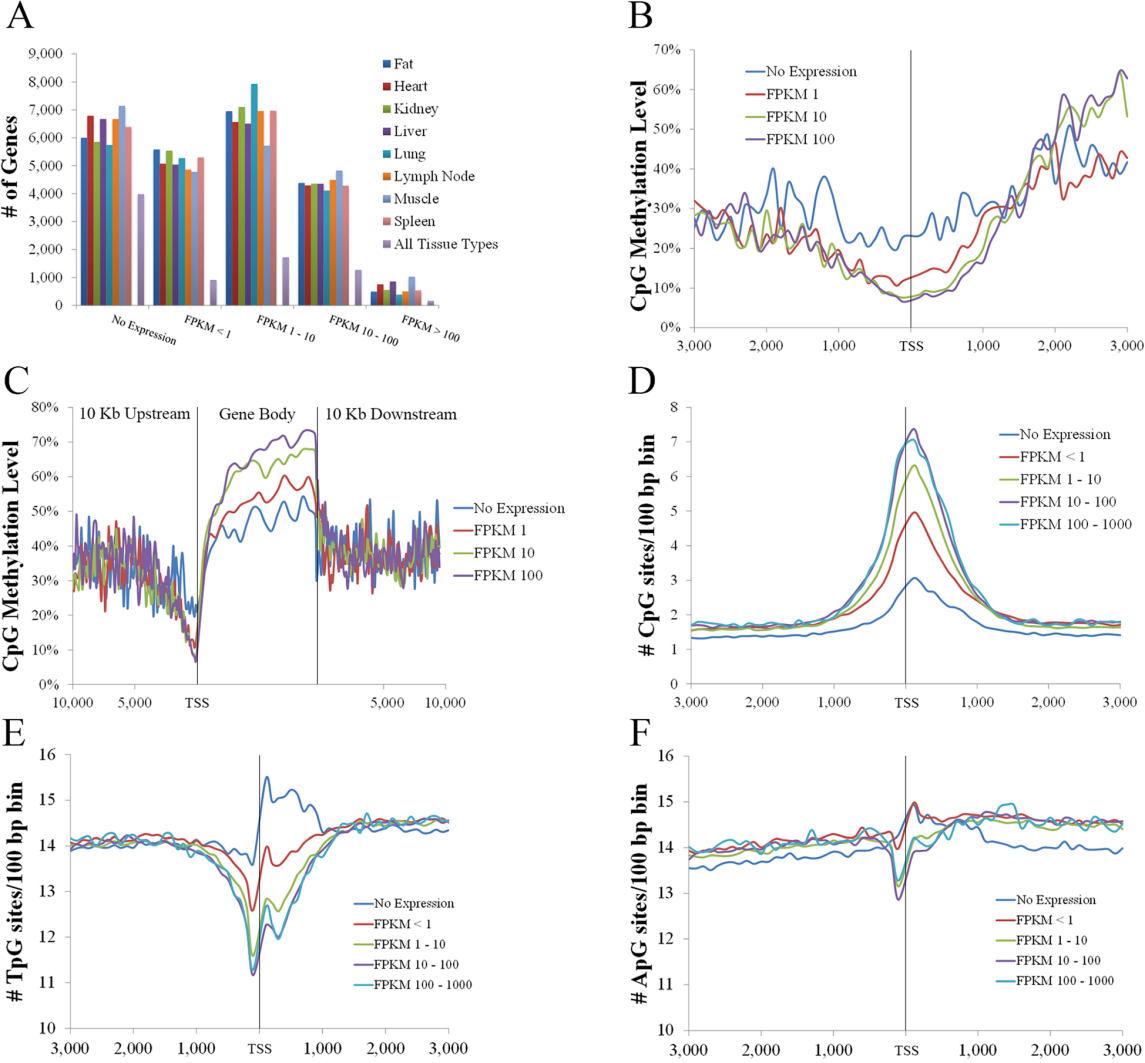
CpG Methylation and density are correlated with gene expression. **a** Gene expression profile of each tissue type and number of genes expressed at each level in all tissues. **b** Negative correlation between CpG methylation and gene expression at TSS. **c** Positive correlation between CpG methylation and gene expression within gene bodies. **d** Positive correlation between CpG density and gene expression at TSS. **e** Negative correlation between gene expression and TpG density at TSS. **f** Negative correlation between gene expression and ApG density at TSS. X axis represents distances in bp

Similar genome wide expression levels were observed between all tissue types, with an average of 27.57 % of genes displaying no expression (Fig. 4a). The majority of profiled genes were predominantly expressed at levels below 100 fragments per kilo bases of exons per million mapped reads (FPKM; 69.61 %), with genes expressed at levels above 100 FPKM representing an average of 2.82 % in all tissues (Fig. 4a). Despite the similarity in the number of genes detected at each expression level, pairwise comparisons revealed on average, between 48 and 68 % of genes expressed at a given level in one tissue were found to be expressed at the same level in another tissue, with < 15 % shown to be expressed at the same level in all tissues, highlighting the differences in gene expression between tissue types (Fig. 4a). This finding was in contrast to a high number of genes found to have no expression in pairwise comparisons (average of 84.28 %) and each of the eight tissue types (44.86 % of non-expressed genes; Fig. 4a).

DNA methylation has been associated with gene expression in a variety of species and cell types [59–61]. As the number of genes with expression levels above 100 FPKM is on average an order of magnitude lower than that for the other expression levels, this range was excluded from further methylation analysis. Consistent with previous reports [59], this study revealed a negative correlation between TSS CpG methylation and gene expression (Spearman’s rho −0.20 to−0.25, *p* < 1×10^−15^; Fig. 4b, Additional file 15: Figure S3), and a visible but statistically weak positive correlation between gene body CpG methylation and expression for all tissue types (Spearman’s rho 0.05 − 0.12, *p* < 0.01; Fig. 4c, Additional file 16: Figure S4).

In addition to the negative correlation between TSS CpG methylation and expression, TSS CpG methylation and density were also negatively correlated (Fig. 2c, e). These results led to the hypothesis that TSS CpG density is positively correlated with gene expression. To test this hypothesis, TSS CpG density was compared to gene expression for all tissue types. As hypothesized, the results revealed a positive correlation between TSS CpG density and gene expression for all tissue types (Spearman’s rho 0.32 − 0.35, *p* < 1×10^−15^; Fig. 4d, Additional file 17: Figure S5), despite the low number of shared genes at each expression level.

It was further hypothesized that the correlation between TSS CpG density and expression is due to the high mutation rate of cytosines in CpG compared to non-CpG context [62], with transition mutations (C to T) being more common than transversions (C to A or G). Therefore, a higher TpG (and to a lesser extent ApG) site density would be expected in the TSS region of lowly compared to highly expressed genes, resulting in negative correlations between expression and the density of these sites at the TSS. As a higher number of GpG sites are likely to be found in GC rich genes, which are known to be highly expressed [63, 64], these sites were not considered for correlation analysis. As hypothesized, the results revealed a negative correlation between TSS TpG density and gene expression (Spearman’s rho −0.23 to−0.25, *p* < 1×10^−15^; Fig. 4e, Additional file 18: Figure S6), in addition to a weaker negative correlation between TSS ApG density and gene expression in all tissues (Spearman’s rho −0.05 to−0.08, *p* < 1×10^−15^; Fig. 4f, Additional file 19: Figure S7), suggesting that the lower TSS CpG density in lowly expressed genes is predominantly due to the deamination of 5-methylcytosine to thymidine over time.

### DNA methylation patterns associated with allelic expression

In addition to changes in methylation associated with expression, previous reports have also detected differential methylation between alleles of genes displaying ASE [65]. While distinguishing differences in methylation patterns between alleles was not within the scope of this project, global methylation patterns of ASE genes were profiled. ASE genes were defined as genes in which one allele was expressed at a significantly higher level than the other (*q* < 0.05). A total of 3,530 genes contained SNP sites and were tested for ASE (see Methods). Of these, 758 demonstrated ASE patterns in at least one tissue type, with an average of 178 genes/tissue (Table 1). Of the 758 ASE genes, 476 displayed tissue specific ASE, resulting in an average of 33.36 % of ASE genes in a given tissue being tissue specific (Table 1). ASE genes were compared to a list of known human (245) and porcine (30) imprinted genes downloaded from the imprinted gene database [66]. Of the known human and porcine imprinted genes, 25 and 4 contained SNPs in our dataset and were assessed for ASE, respectively. Of these genes, 7 human and all porcine imprinted genes showed patterns of ASE in at least one tissue, respectively, demonstrating the ability of the analysis to detect known imprinted genes. Additionally, a total of 64 genes were found to be mono-allelically expressed (expressed from only one allele) in at least one tissue type, with an average of 24 genes/tissue (Table 1). Of the 64 mono-allelic expressed genes, 27 displayed tissue specific mono-allelic expression, resulting in an average of 13.85 % of mono-allelic expressed genes in a given tissue being unique to that tissue (Table 1). This translates to a higher average proportion of tissue specific ASE genes than mono-allelic expressed genes (33.36 and 13.85 %, respectively, *p* < 0.00001; Table 1)

**Table 1:** Number of ASE and mono-allelic genes detected in each tissue

	ASE	Unique ASE	Mono-allelic	Unique Mono-allelic
Fat	225	75	25	3
Heart	138	43	24	2
Kidney	217	73	24	3
liver	210	80	21	2
Lung	195	68	25	5
Lymph Node	135	35	27	3
Muscle	135	53	25	6
Spleen	172	49	24	3

As imprinted genes have been shown to be essential for metabolic, regulatory, and developmental processes [67], the functional characteristics of porcine ASE genes were assessed using GO term and KEGG pathway enrichment analysis. GO terms and KEGG pathways containing significantly more ASE genes than expected by chance (*q* < 0.05) were considered enriched in each tissue. The 282 genes displaying ASE in at least 2 tissues were used for GO term and KEGG pathway analysis in order to limit any tissue specific bias. A total of 17 GO terms and 9 KEGG pathways were enriched for ASE genes (Additional file 20: Table S13). Of the enriched GO terms, 6 related to the regulation of cell death and apoptosis, 6 to response to oxygen species and oxidative stress, 4 to the formation of protein oligomers, and 1 related to the regulation of cell proliferation (Additional file 20: Table S13). Of the enriched KEGG pathways, the majority were involved in metabolism, with 2 related to genetic information processing (1 at the transcriptional and 1 at the translational level; Additional file 20: Table S13). Overall the results suggest that porcine ASE genes are involved in metabolic and regulatory processes.

**Fig. 5:**
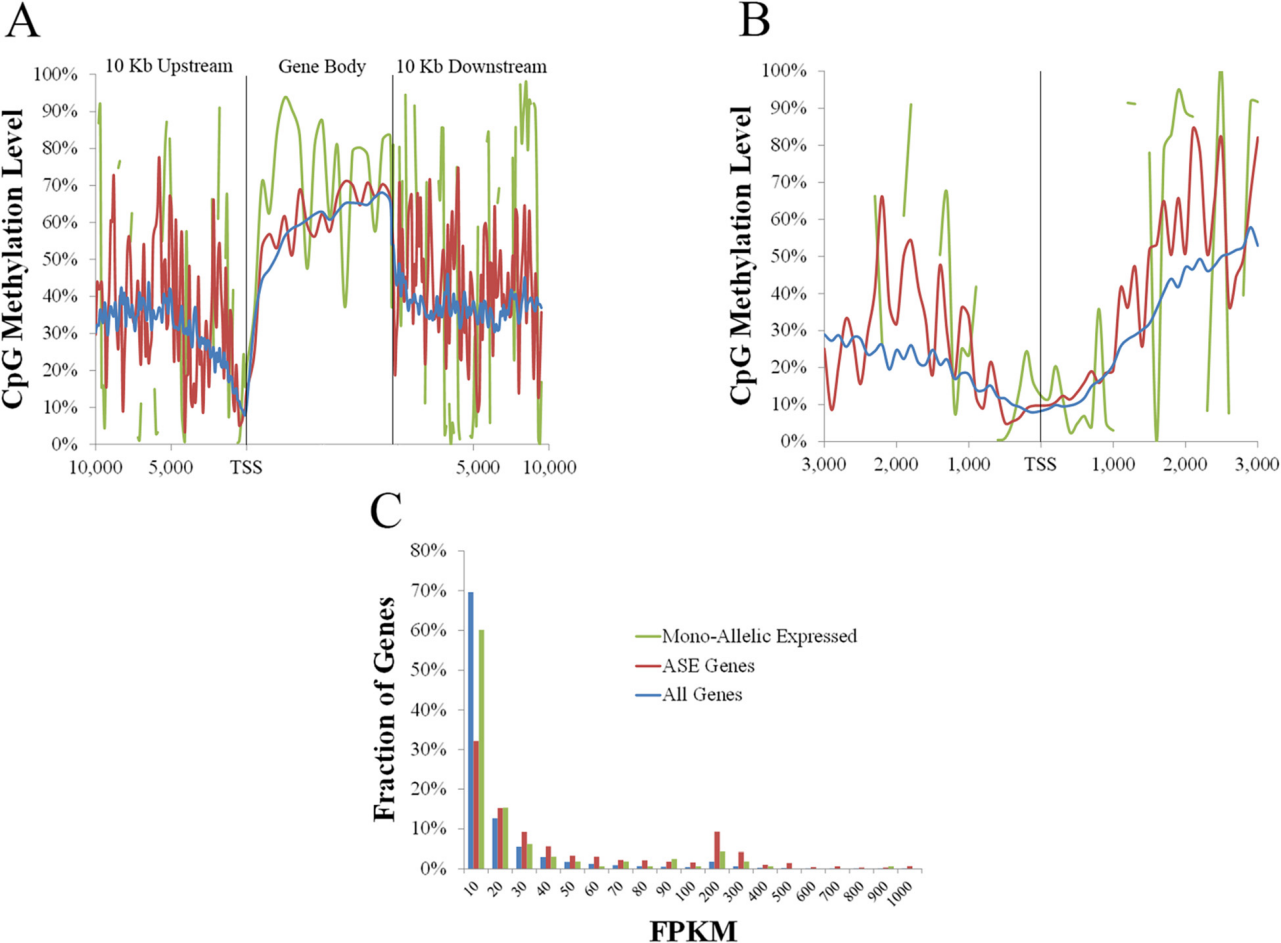
CpG methylation of ASE genes. **a** CpG methylation in gene bodies of ASE and mono-allelic expressed genes compared to all expressed genes. **b** Differences in CpG methylation at TSS of ASE and mono-allelic expressed genes compared to all expressed genes. X axis represents distances in bp. **c** Expression profile of ASE and mono-allelic expressed genes compared to all expressed genes

In order to determine whether differences in CpG methylation were apparent in these genes, both ASE and mono-allelic expressed genes were profiled and compared to the average level for all expressed genes (Fig. 5). Although the average methylation levels across all tissue types revealed hypermethylation within gene bodies of ASE and mono-allelic expressed genes (Fig. 5a, b), expression profiles also revealed on average higher expression levels in ASE (median FPKM of 26.49) and mono-allelic expressed genes (10.59) compared to all genes (4.08; Fig. 5c). Therefore, observed differences in CpG methylation between ASE, mono-allelic, and all expressed genes can be explained by differences in expression.

### Independent validation of gene expression and DNA methylation patterns

In order to validate the gene expression and DNA methylation patterns reported, RRBS and RNA-seq was performed on liver, muscle, and spleen from an unrelated adult male Duroc (validation dataset) and compared to the results from this study. The log2 fold changes in gene expression from the 10 most up- and downregulated genes between each tissue comparison from the adult female Duroc (liver vs. muscle, liver vs. spleen, and spleen vs. muscle) were compared to the differences observed for the same genes (60) in the validation dataset. The results revealed the same directional change in expression between tissues for all genes tested (Additional file 21: Figure S8a, b, and c). In addition, the log2 fold changes were highly correlated between the adult female Duroc and the validation dataset (Spearman’s Rho 0.899, *p* < 1×10^−15^; Additional file 21: Figure S8d), further validating the gene expression results presented here.

Validation of the DNA methylation results was performed by determining the 10 CpG sites displaying the largest increase and decrease in methylation level for each tissue comparison from the adult female Duroc and comparing them to the differences observed for the same sites in the validation dataset. The results revealed the same directional change in methylation level between tissues for 56 of the 60 sites tested (Additional file 22: Figure S9a, b, and c). Three of the 4 sites that did not display the same directional change were unmethylated in both tissues in the validation dataset, while the other displayed an opposing directional change when comparing spleen and liver (64.9 % lower and 14.04 % higher methylation in the adult female Duroc and validation dataset, respectively). It is also worth noting that 3 of the 4 differing sites were from comparisons involving the spleen, which is more likely to have varied methylation due to environmental differences resulting in differing immune system status between these individuals. In addition, the log2 fold changes in methylation were highly correlated between the adult female Duroc and the validation dataset (Spearman’s Rho 0.831, *p* < 1×10^−15^; Additional file 22: Figure S9d), further validating the DNA methylation results presented here.

## Discussion

As the evidence for linkage between epigenetics and health continues to grow, there is an increasing need for a better understanding of how these patterns affect cellular processes, as well as how they can be influenced by environmental signals. While profiling these patterns in human cells is essential to understanding the mechanisms behind these diseases and how they contribute to human health, an understanding of the functional similarities and differences between epigenetic patterns in humans and model organisms used to study human disease is critical. This project utilized tissues from the same individual chosen for production of the swine reference genome (Sscrofa10.2), providing transcriptional and DNA methylation datasets for the biomedical community directly relatable to current genomic resources.

One disadvantage of this study was the inability to infer cell type specific methylation patterns, as DNA methylation patterns are known to vary across cell types [68]. However, while the majority of human samples profiled in studies such as the human ENCODE project consist of cell lines, tissue specific methylation patterns are useful to the swine biomedical community due to the current lack of standardized commercially available porcine cell lines. Indeed, while there are > 1,100 human cell lines available from ATCC [69], only 8 porcine cell lines (3 kidney epithelial, 3 alveolar macrophage, and 2 fibroblast cell lines) are currently available. Furthermore, altered methylation patterns have been observed in transformed cell lines as well as lines with high numbers of passages [70–73]. Therefore the results presented here are likely to be more representative of *in vivo* states than those derived from transformed cell lines with high passage rates.

When investigating genome-wide DNA methylation patterns, it is important to consider which technique is most appropriate for a given study. Whole genome bisulfite sequencing (WGBS) is quickly becoming the standard technology used to detect genome-wide methylation patterns in humans and mice due to its ability to cover the vast majority of the CpG and non-CpG sites in a genome. Therefore the ability of this study to investigate genome-wide DNA methylation patterns is limited to the subset of sites located within the RRBS target regions. Additionally, the observed methylation patterns in these regions tend to underestimate genome-wide methylation levels and the frequency of methylated CpG sites, as RRBS targets CpG rich regions throughout the genome, and methylation is negatively correlated with CpG density. However, the increased costs associated with the use of WGBS, such as the need to sequence the entire genome to an appropriate depth compared to sequencing < 2 % of the genome here, limits its use in this and many other studies. Additionally, the consistent enrichment of CpG sites of interest, such as those located within CGIs, was demonstrated previously in a porcine study using RRBS to assess altered intestinal DNA methylation patterns associated with preterm birth and development [22]. This study further demonstrates the ability of RRBS to target CpG sites of interest in pigs, with 73 % of the CpG sites covered across tissue types located within either CGIs or gene regions. Additionally, the distribution of covered CpG sites revealed a bimodal distribution typical of previous human RRBS studies [55]. These patterns, as well as the confirmation of tissue specific DNA methylation changes in the validation dataset, confirm RRBS is effective in targeting and detecting relevant methylation levels in CpG rich regions of interest in pigs, further validating it as a suitable technique for studying porcine genome-wide methylation patterns.

Given the fact that CpG dinucleotides mutate at higher rates due to the deamination of 5-methylcytosine to thymidine, it is important to remove SNP sites before analysis of methylation data, as their inclusion in downstream analyses could result in incorrect methylation estimates, ultimately biasing results, particularly for studies in which differential methylation analysis is performed. In this study the ability of a targeted control library to detect SNPs at CpG sites was assessed. This targeted control dataset was able to cover a significantly higher percentage of the CpG sites used for methylation analysis than the WGS dataset (86.68 % compared to 29.33 %), despite the high average coverage (25×) of the WGS dataset. While surprising, the low coverage of CpG rich regions by the WGS dataset is most likely due to the GC-bias of Illumina sequencing data. Therefore, the utilization of targeted control datasets as described here provides effective detection of SNPs at CpG sites targeted by RRBS studies compared to traditional WGS datasets used for SNP detection. However, as the total number of SNPs detected by the targeted control dataset represents a relatively small percentage of the total CpG sites assessed for methylation analysis (0.67 %), use of a control dataset is probably not necessary for genome wide studies such as this, but could be important in studies performing site specific methylation comparisons. It is also important to note that in addition to directly effecting detected methylation levels at CpG sites, SNPs at CpG sites also have the potential to affect methylation levels of surrounding sites by altering the GC content and CpG density of the region. While not investigated here, targeted control libraries could be used to investigate the effects of SNPs on regional methylation levels in studies consisting of individuals with diverse variation.

The observed increased CpG methylation level, as well as the increased proportion of methylated (>10 %) and decreased proportion of unmethylated CpG sites on the X chromosome compared to the autosomes are similar to patterns observed in human female samples [74], suggesting CpG methylation is important in porcine X chromosome inactivation. Additionally, no significant differences in non-CpG methylation distribution were observed between the X chromosome and the autosomes, suggesting non-CpG methylation does not play a role in X-inactivation in adult somatic tissues. While the majority of the observed methylation patterns were consistent with reported patterns in human cells [55], the higher CpG methylation within introns compared to exons is in contrast to reports in human cell lines [60, 75]. At this time it is unclear whether the opposing patterns observed between porcine tissues and human cell lines is due to differences in the evolution of these patterns in the two species, differences in cell line vs tissue patterns, or both. However, these results ultimately support previous reports suggesting a role of CpG methylation in defining intron exon boundaries and regulating splicing [76, 77].

Recently non-CpG methylation has been demonstrated to be correlated with gene expression in embryonic stem cells, induced pluripotent stem cells, and oocytes of many mammalian species [55, 78, 79], as well as in mammalian neuronal tissue [80, 81]. Additionally, low levels of non-CpG methylation have also been reported in human and porcine non-neuronal somatic tissues, with no known functional relevance [22, 55]. Here we report non-CpG methylation for all eight somatic tissue types distributed in a non-random fashion, suggesting a relationship between non-CpG methylation and genomic features. The increased non-CpG methylation within CGS compared to CGIs, and introns compared to exons was similar to the pattern observed for CpG methylation, albeit at a much lower level. While these differences were not significant in all tissue types, this trend was observed for each tissue. Reduced non-CpG methylation toward the 5’ end of genes was also detected, with the lowest level found at the TSS. While similar correlations have been reported in human pluripotent cells [55], these patterns have not been previously reported in somatic non-neuronal tissues. Although it has been reported that RRBS datasets are capable of detecting a representative sample of non-CpG sites throughout the human genome [55], it is important to note that the sites covered in this study represent only 0.21 % of all non-CpG sites in the pig genome, and therefore more extensive studies are required to confirm whether these results are representative, and whether non-CpG methylation plays a functional role in these tissues.

Although DNA methylation patterns are known to form tissue specific patterns as cells undergo differentiation [82], the results from this study revealed similar genome-wide DNA methylation patterns across multiple tissue types in relation to CGI, CGS, TSS, and gene bodies. It is important to note that while the genomic methylation patterns in relation to genic regions were similar for all eight tissues, the resulting cluster analysis suggests that tissue specific patterns are still present. While tissues with similar functions, such as muscle (skeletal muscle and heart) and immune tissues (lymph node and spleen) did not cluster together when assessing CpG methylation, the immune tissues did cluster together when assessing non-CpG methylation, highlighting the differential clustering of tissue types when assessing CpG and non-CpG methylation. Additionally, the smaller height observed for the CpG cluster suggests that tissue types are more similar in terms of CpG methylation, and that there are differences in how these patterns vary across tissue types. However, the lack of CpG methylation based tissue clustering by function or cell lineage is surprising, and could be due to a number of factors including the use of tissue samples as opposed to purified cell lines, or the reduced nature of the RRBS datasets. Future profiling of these tissues in additional individuals will help provide further insights into the relatedness of these tissues in terms of CpG and non-CpG methylation, and how they compare to known patterns in humans.

As the number of studies reporting CpG methylation associated with gene expression continues to increase, it is becoming increasingly apparent that the genomic location of CpG sites is critical in determining what effect methylation has on expression. Both a negative correlation between TSS CpG methylation and gene expression, as well as a positive correlation between gene body CpG methylation and gene expression were observed. While the observed positive correlation between gene body CpG methylation and gene expression was statistically weak, this result could be due to the lower average coverage of CpG sites within gene body regions (2.18 %) compared to TSS (10.17 %), resulting in lower accuracy when correlating CpG methylation with expression of individual genes. Overall, these results are consistent with previous reports in human somatic tissues [59], supporting the hypothesis that porcine genomic CpG methylation displays similar genomic distribution and functional patterns as human cells.

Consistent with previous reports in humans [57, 58], an increase in CpG density at the TSS of pig genes, as well as a negative correlation between CpG density and methyaltion at TSS and CGIs was observed. Surprisingly, the correlation between CpG density and methylation was not found to be consistent across the genome, with a positive correlation observed in CGS of all tissue types. This result is surprising, as it suggests the relationship between CpG density and methylation is dependent on genomic context, and can vary within relatively small regions, as CGIs and CGS are directly adjacent to one another throughout the genome. However, as the CGS tested for correlations between CpG density and methylation had on average a lower CpG density than CGIs and TSS tested (6.3 CpG sites/100 bp compared to 18.7 and 17.4, respectively), it is possible that the negative correlation between CpG density and methylation is only observable when including regions above a given CpG density threshold.

In addition, this study also reports a positive correlation between TSS CpG density and expression apparent for all eight tissues. While core sets of genes were expressed at similar levels in all tissues, these genes represented a relatively low percentage found at each expression level. Therefore it was surprising that this pattern was apparent in all tissues. Cytosines in CpG compared to non-CpG context mutate at a higher rate, with transition mutations being more common than transversions due to the deamination of 5-methylcytosine to thymidine [53, 62], resulting in the conversion of CpG to TpG sites. Therefore, this paper proposes that the reduced number of CpG sites at TSS of lowly expressed genes is due to the high CpG site mutation rate in these regions over time, predominantly due to the deamination of 5-methylcytosine to thymidine. This theory is supported by the observed negative correlation between TSS TpG density and gene expression, and the weaker negative correlation between TSS ApG density and gene expression. Combined with the known alteration of methylation patterns in relation to environmental stimuli, this mechanism provides a way in which CpG methylation could result in more permanent changes to lower gene expression.

Genetic variation plays a major role in adaptive evolution, as demonstrated by the rapid evolution of highly expressed genes in the brains of dogs linked to the evolution of dog-specific characteristics [83]. In addition to changes in nucleotide sequence, many studies have found gene expression differences in multiple brain regions associated with tameness and other domestication-based phenotypes between domesticated and wild species [83–85]. Gene expression changes like these are seen in many species adapting to a variety of environmental changes [86, 87]; however, the mechanisms by which these changes occur are not fully understood. While genetic variation at transcription-factor binding sites are associated with regulatory changes and phenotypic variation in many species [87], alternative mechanisms, like the proposed mutation of CpG sites at TSS, may also be responsible for long and short term regulation of genes in response to environmental changes. This would make DNA methylation important for adaptive evolution, as it is believed that regulatory changes in gene expression play a particularly important role in the evolutionary process [3, 4].

## Conclusions

This study confirmed the presence of porcine CpG methylation patterns similar to those previously demonstrated for humans and mice, as well as confirmed the functional aspects of porcine CpG methylation, observing a negative correlation with gene expression at TSS and a positive correlation within gene bodies. Additionally, a positive correlation between TSS CpG density and gene expression was observed in all tissue types. This suggests that increased mutation rates at CpG sites play a significant role in adaptive evolution by reducing CpG density at TSS over time, resulting in higher methylation levels in these regions and more permanent changes to lower gene expression. Our data support that this occurs predominantly through deamination of methylated cytosine to thymidine, resulting in the replacement of CpG with TpG sites in these regions, as indicated by the increased TSS TpG density observed in non-expressed genes, resulting in a negative correlation between expression and TSS TpG density. Further studies are required to investigate the similarities in methylation levels between humans and pigs for specific genomic regions known to affect disease progression, the differences observed in intron and exon methylation patterns between pig tissues and human cell lines, and the proposed adaptive evolutionary role of CpG methylation. In conclusion, porcine CpG methylation levels were similar to those reported for other mammals, further supporting pigs as viable models for studying the relationship between DNA methylation and environmental insults, as well as the diagnosis, detection, and treatment of human diseases with known links to aberrant DNA methylation patterns.

## Availability of supporting data

The data sets supporting the results of this article are available in the European Nucleotide Archive and are available under accession number PRJEB8784 (www.ebi.ac.uk/ena/data/view/PRJEB8784). Additionally, track hubs containing expression and methylation data for all tissues are available for viewing on the Ensembl and UCSC genome browsers as part of the FAANG initiative [88] (www.faang.org).

## Additional files

Additional file 1: Table S1. Adult female Duroc RRBS and RNA-seq read depths. (XLSX 8 kb) Additional file 2: Table S2. Validation dataset RRBS and RNA-seq read depths. (XLSX 8 kb) Additional file 3: Figure S1. Read distribution of WGS and targeted control datasets. Read depth distribution of WGS dataset for **a** all genomic positions and **b** high confidence CpG sites. Read depth distribution of control dataset for **c** all genomic positions and **d** high confidence CpG sites. (PNG 9582 kb) Additional file 4: Table S3. CpG site coverage and methylation level in relation to genomic features. * denotes percent of sites covered out of total in size fraction. (XLSX 11 kb) Additional file 5: Figure S2. Genomic distribution of covered CpG sites. The distribution of covered CpG sites in relation to **a** CGIs and CGS, and **b** genic regions. **c** Overlap of covered CpG sites between CGIs and gene regions. **d** Bimodal distribution of CpG sites in relation to methylation level. The distribution of covered **e** CpG and **f** non-CpG sites per chromosome in relation to chromosomal CpG density. (PNG 16855 kb) Additional file 6: Table S4. Proportion of unmethylated and methylated (>10 %) CpG sites across tissues. (XLSX 9 kb) Additional file 7: Table S5. CpG methylation level across chromosomes. (XLSX 10 kb) Additional file 8: Table S6. Proportion of unmethylated CpG sites across chromosomes. (XLSX 11 kb) Additional file 9: Table S7. Proportion of methylated (>10 %) CpG sites across chromosomes. (XLSX 11 kb) Additional file 10: Table S8. Methylation level, proportion unmethylated, and methylated (>10 %) CpG sites across chromosomes in male validation dataset. (XLSX 11 kb) Additional file 11: Table S9. Non-CpG site coverage and methylation level in relation to genomic features. * denotes percent of sites covered out of total in size fraction. (XLSX 11 kb) Additional file 12: Table S10. Methylation at CHG and CHH sites. (XLSX 10 kb) Additional file 13: Table S11. Proportion of unmethylated non-CpG sites across chromosomes. (XLSX 11 kb) Additional file 14: Table S12. Proportion of methylated (>1 %) non-CpG sites across chromosomes. (XLSX 11 kb) Additional file 15: Figure S3. Negative correlation between TSS CpG methylation and gene expression in all tissues. CpG methylation levels in relation to gene expression at TSS for **a** fat, **b** heart, **c** kidney, **d** liver, **e** lung, **f** lymph node, **g** muscle, and **h** spleen. X axis represents distances in bp. (PNG 15538 kb) Additional file 16: Figure S4. Positive correlation between gene body CpG methylation and gene expression in all tissues. CpG methylation levels in relation to gene expression across gene bodies for **a** fat, **b** heart, **c** kidney, **d** liver, **e** lung, **f** lymph node, **g** muscle, and **h** spleen. X axis represents distances in bp. (PNG 15591 kb) Additional file 17: Figure S5. Positive correlation between TSS CpG density and gene expression in all tissues. CpG density in relation to gene expression at TSS for **a** fat, **b** heart, **c** kidney, **d** liver, **e** lung, **f** lymph node, **g** muscle, and **h** spleen. X axis represents distances in bp. (PNG 13691 kb) Additional file 18: Figure S6. Negative correlation between TSS TpG density and gene expression in all tissues. Gene expression in relation to TpG density at TSS for **a** fat, **b** heart, **c** kidney, **d** liver, **e** lung, **f** lymph node, **g** muscle, and **h** spleen. X axis represents distances in bp. (PNG 14673 kb) Additional file 19: Figure S7. Negative correlation between TSS ApG density and gene expression in all tissues. Gene expression in relation to ApG density at TSS for **a** fat, **b** heart, **c** kidney, **d** liver, **e** lung, **f** lymph node, **g** muscle, and **h** spleen. X axis represents distances in bp. (PNG 14673 kb) Additional file 20: Table S13. GO terms and KEGG pathways enriched for ASE. (XLSX 10 kb) Additional file 21: Figure S8. Same directional gene expression changes across tissues in the adult female Duroc and validation datasets. Expression differences of the 10 most up- and downregulated genes between each tissue comparison from the adult female Duroc compared to the differences observed for the same genes in the validation dataset. **a** Log2 fold change differences in expression between liver and spleen, **b** liver and muscle, and **c** spleen and muscle. **d** Correlation between log2 fold changes in the adult female Duroc and validation dataset for all 3 tissue comparisons. (PNG 9535 kb) Additional file 22: Figure S9. Differences in CpG site methylation across tissues in the adult female Duroc and validation datasets. Methylation differences of the 10 most hyper and hypomethylated CpG sites between each tissue comparison from the adult female Duroc compared to the differences observed for the same sites in the validation dataset. **a** Methylation level differences at CpG sites between liver and muscle, **b** spleen and muscle, and **c** liver and spleen. **d** Correlation between log2 fold methylation changes in the adult female Duroc and validation dataset for all 3 tissue comparisons. (PNG 9329 kb)

## Abbreviations

ASE: Allele Specific Expression
CGIs: CpG Islands
CGS: CpG Island Shores
FPKM: Fragments Per Kilo Bases of Exons Per Million Mapped Reads
gDNA: Genomic DNA
RRBS: Reduced Representation Bisulfite Sequencing
TSS: Transcription Start Site
WGS: Whole Genome Sequencing
WGBS: Whole Genome Bisulfite Sequencing
