# Supplementary figures and images for: Adult porcine genome-wide DNA methylation patterns support pigs as a biomedical model
Source: BMC Genomics. 2015 Oct 5;16:743. doi: 10.1186/s12864-015-1938-x (PMC4594891; doi:10.1186/s12864-015-1938-x)

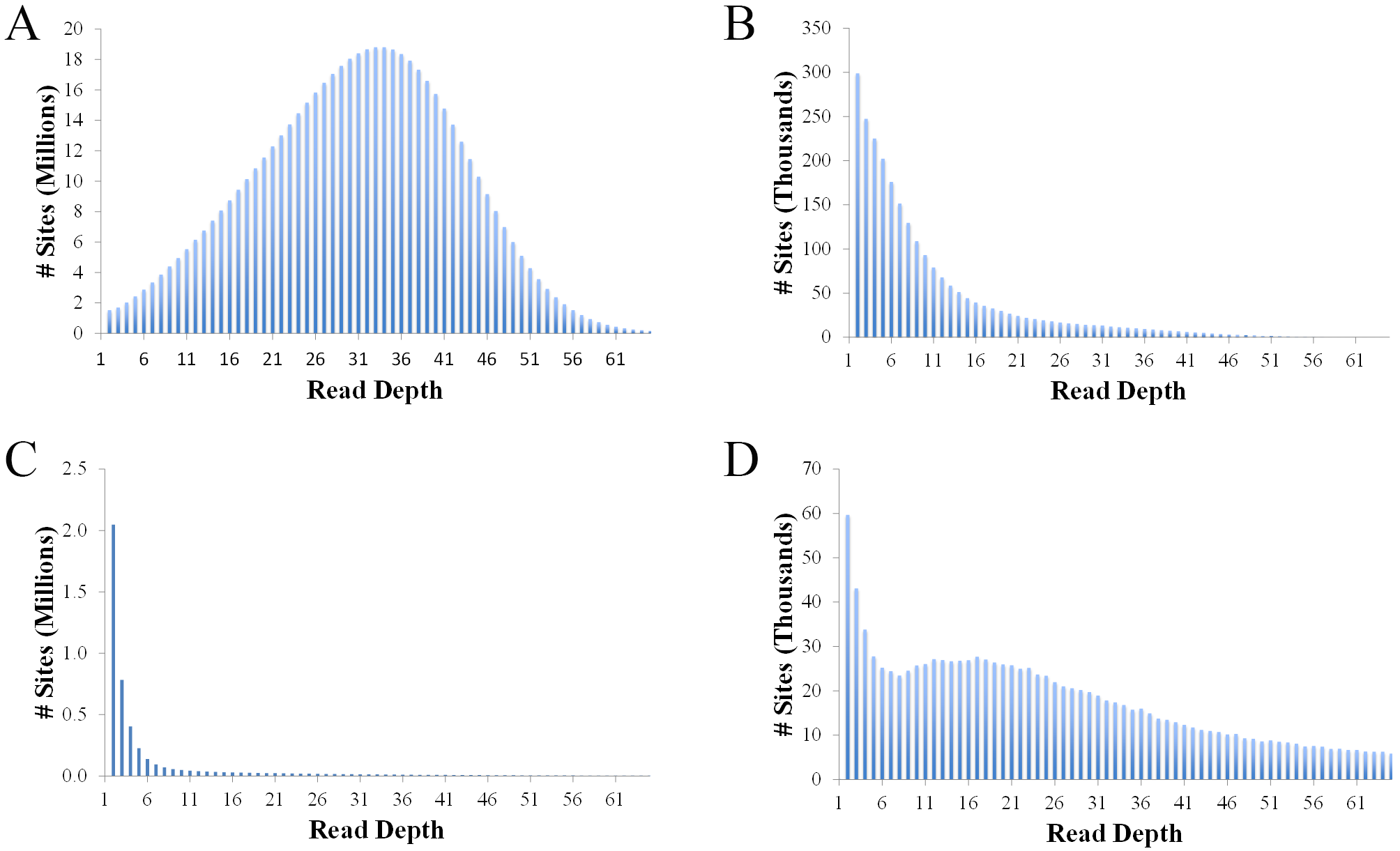

Supplement: Additional file 3: Figure S1. — Read distribution of WGS and targeted control datasets. Read depth distribution of WGS dataset for a all genomic positions and b high confidence CpG sites. Read depth distribution of control dataset for c all genomic positions and d high confidence CpG sites. (PNG 9582 kb) [file 12864_2015_1938_MOESM3_ESM.png]

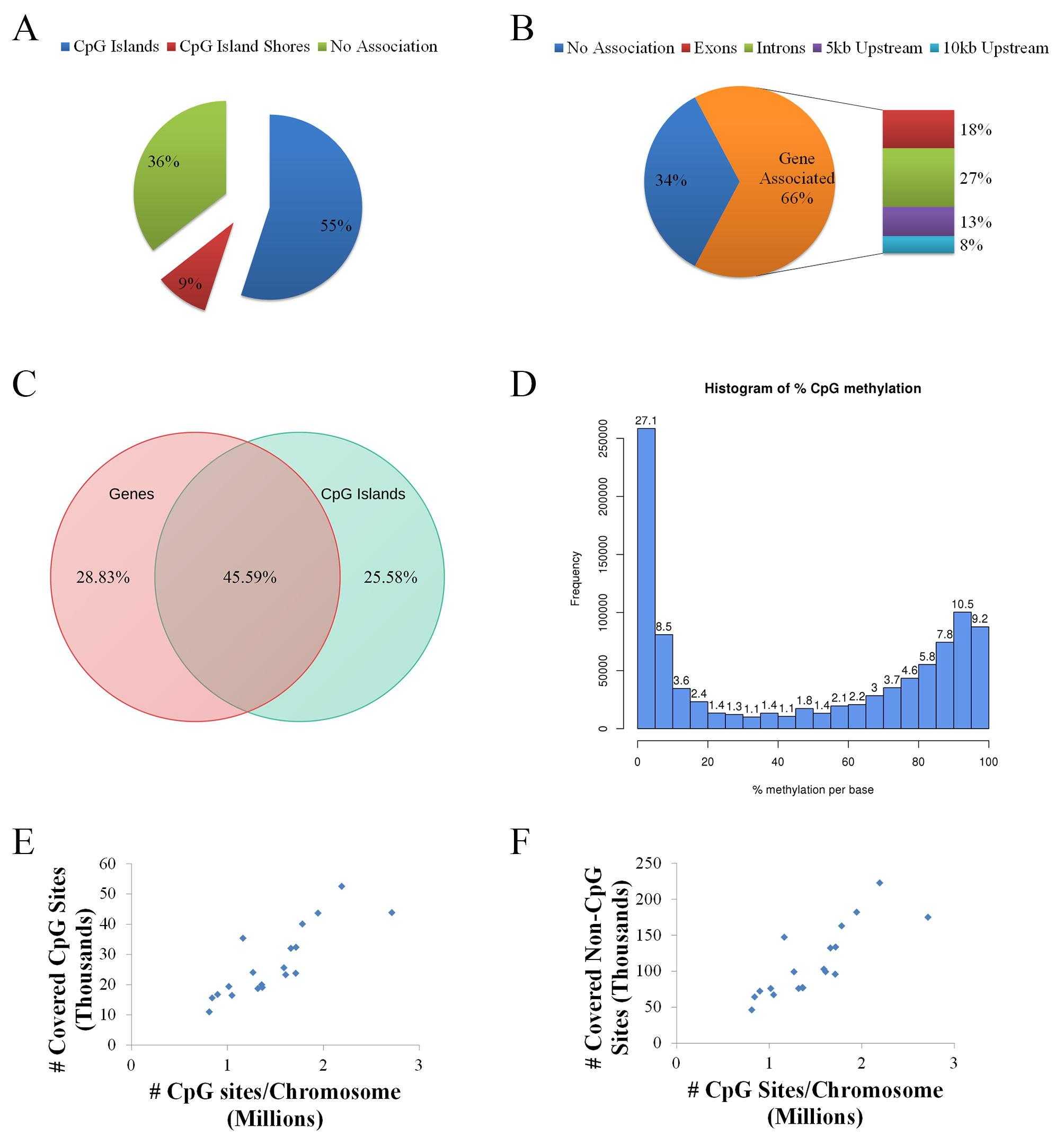

Supplement: Additional file 5: Figure S2. — Genomic distribution of covered CpG sites. The distribution of covered CpG sites in relation to a CGIs and CGS, and b genic regions. c Overlap of covered CpG sites between CGIs and gene regions. d Bimodal distribution of CpG sites in relation to methylation level. The distribution of covered e CpG and f non-CpG sites per chromosome in relation to chromosomal CpG density. (PNG 16855 kb) [file 12864_2015_1938_MOESM5_ESM.png]

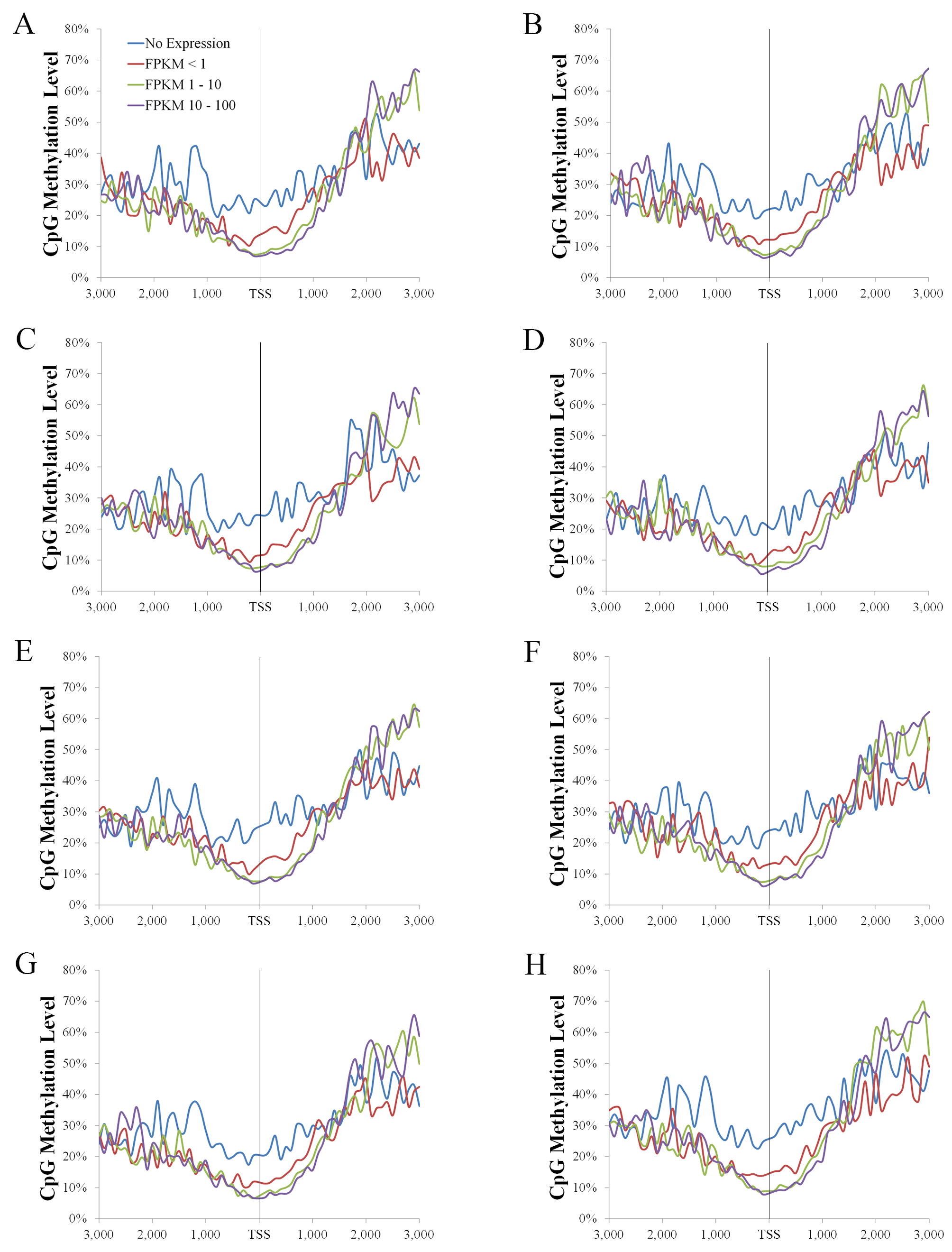

Supplement: Additional file 15: Figure S3. — Negative correlation between TSS CpG methylation and gene expression in all tissues. CpG methylation levels in relation to gene expression at TSS for a fat, b heart, c kidney, d liver, e lung, f lymph node, g muscle, and h spleen. X axis represents distances in bp. (PNG 15538 kb) [file 12864_2015_1938_MOESM15_ESM.png]

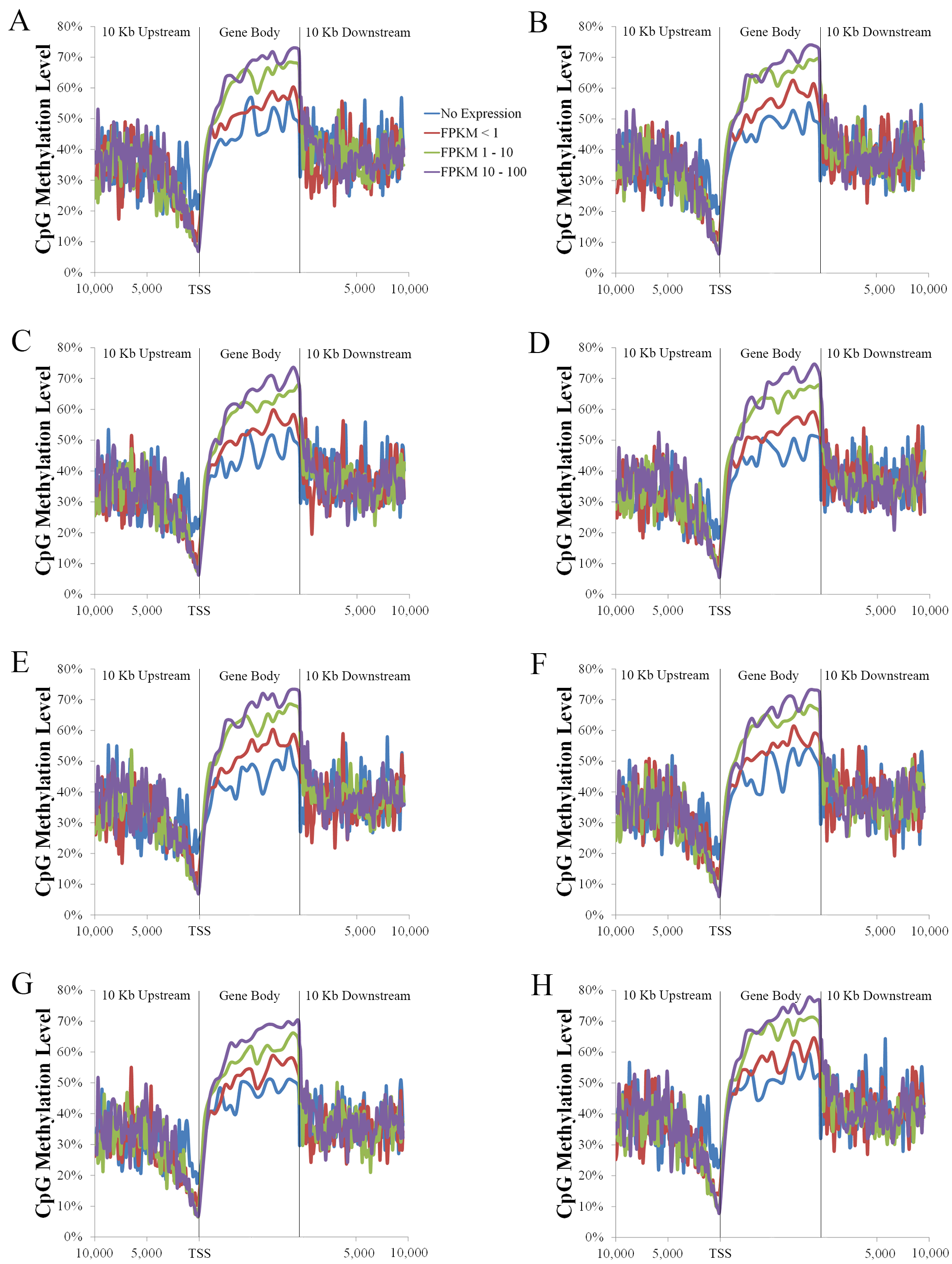

Supplement: Additional file 16: Figure S4. — Positive correlation between gene body CpG methylation and gene expression in all tissues. CpG methylation levels in relation to gene expression across gene bodies for a fat, b heart, c kidney, d liver, e lung, f lymph node, g muscle, and h spleen. X axis represents distances in bp. (PNG 15591 kb) [file 12864_2015_1938_MOESM16_ESM.png]

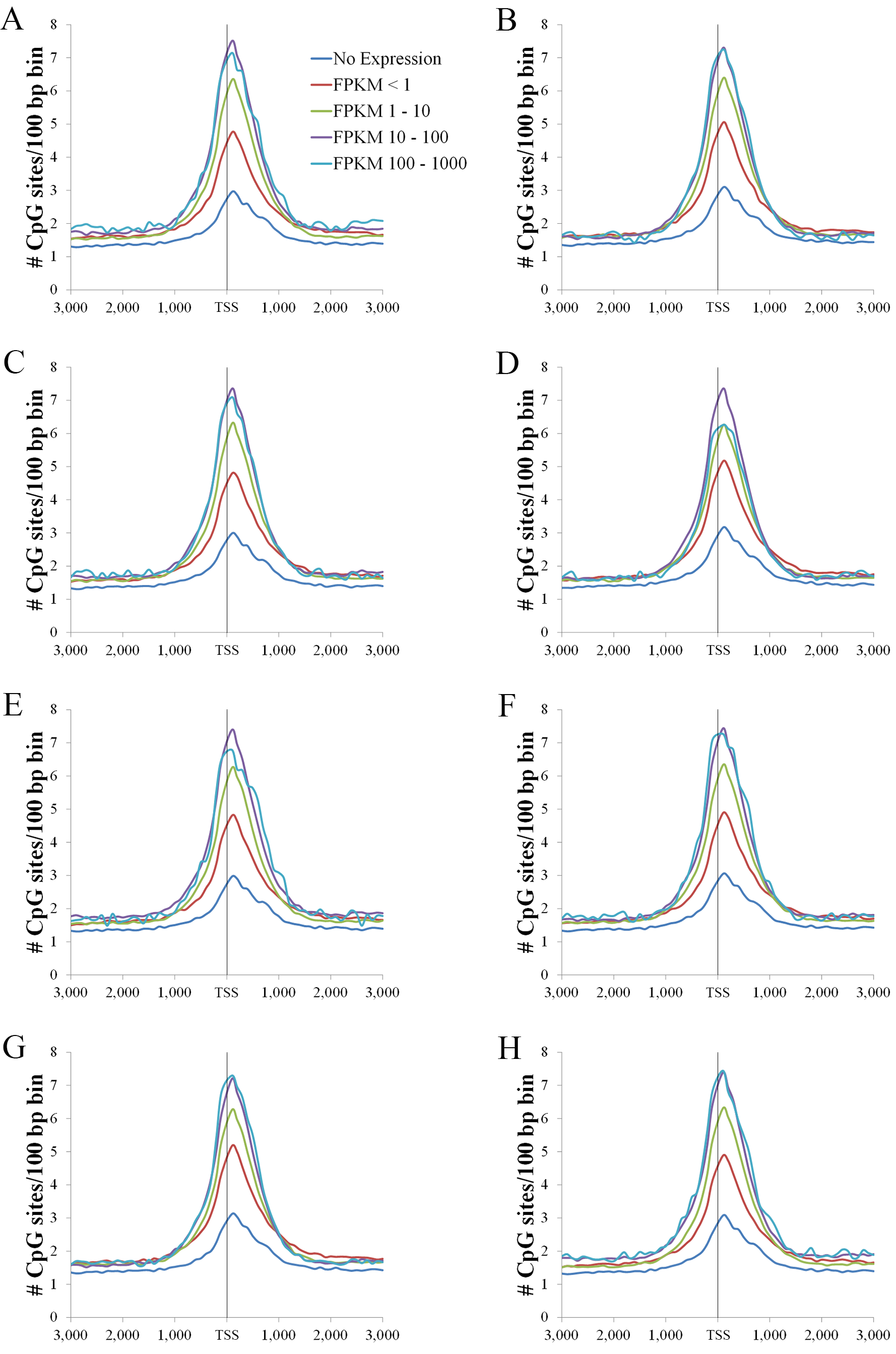

Supplement: Additional file 17: Figure S5. — Positive correlation between TSS CpG density and gene expression in all tissues. CpG density in relation to gene expression at TSS for a fat, b heart, c kidney, d liver, e lung, f lymph node, g muscle, and h spleen. X axis represents distances in bp. (PNG 13691 kb) [file 12864_2015_1938_MOESM17_ESM.png]

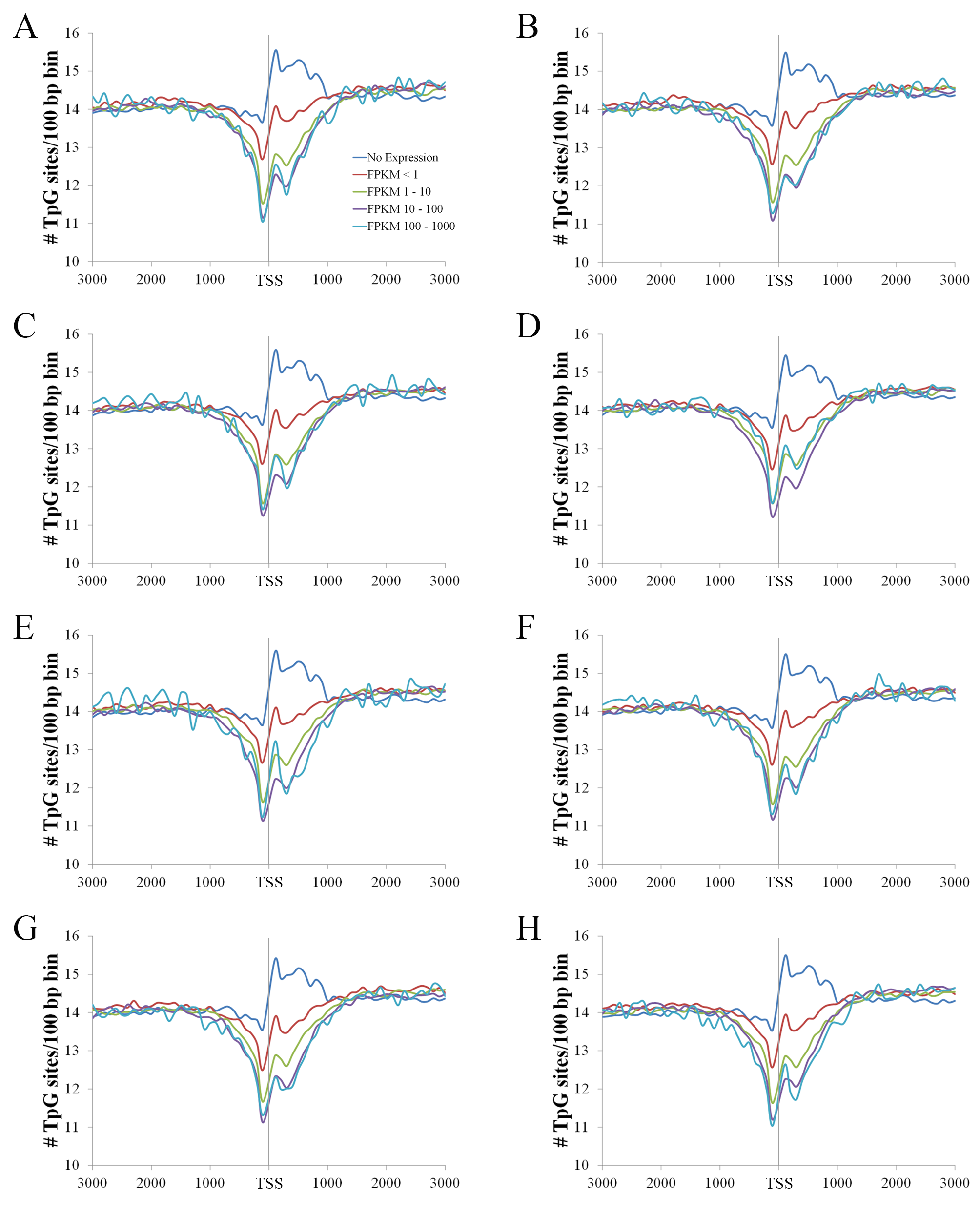

Supplement: Additional file 18: Figure S6. — Negative correlation between TSS TpG density and gene expression in all tissues. Gene expression in relation to TpG density at TSS for a fat, b heart, c kidney, d liver, e lung, f lymph node, g muscle, and h spleen. X axis represents distances in bp. (PNG 14673 kb) [file 12864_2015_1938_MOESM18_ESM.png]

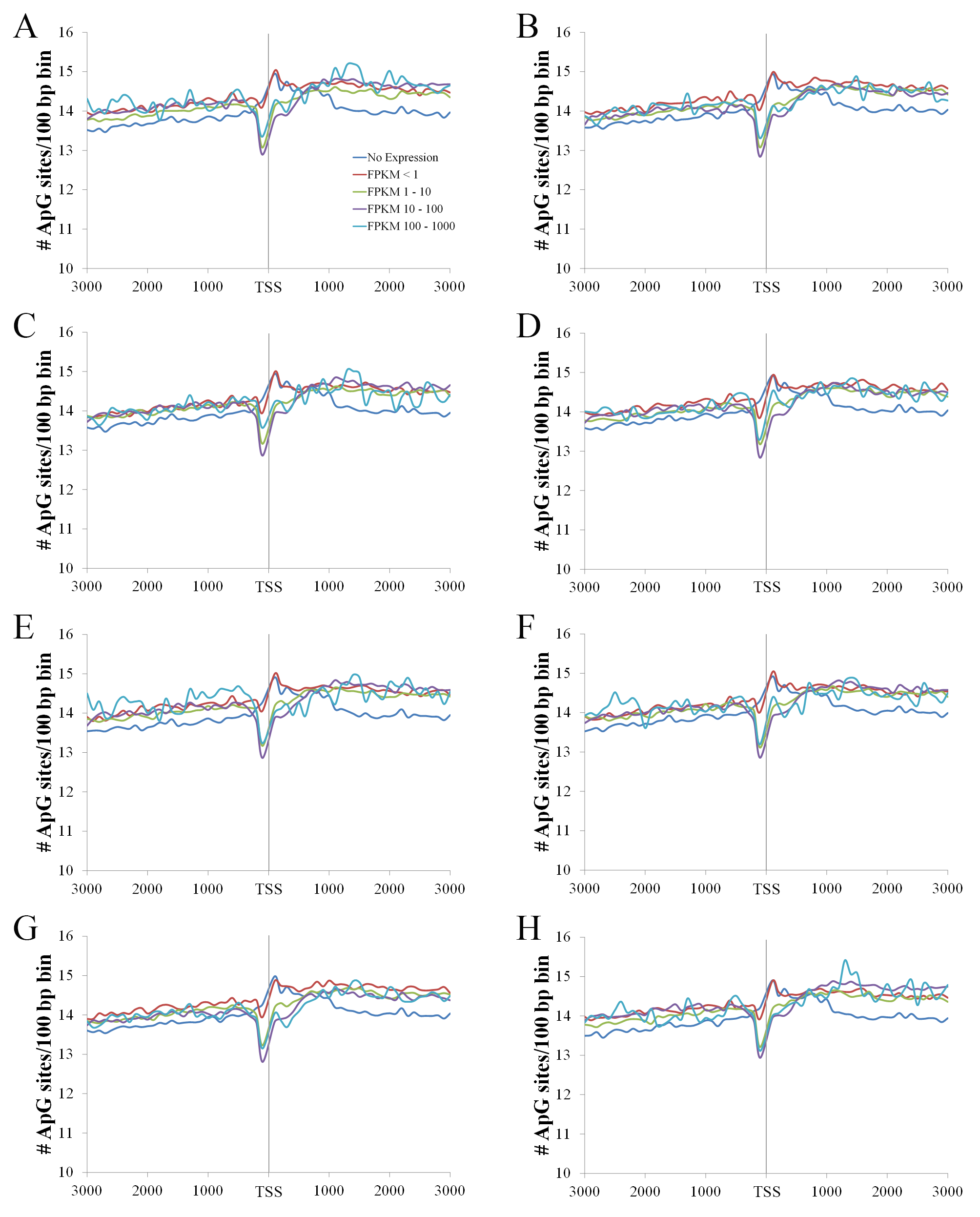

Supplement: Additional file 19: Figure S7. — Negative correlation between TSS ApG density and gene expression in all tissues. Gene expression in relation to ApG density at TSS for a fat, b heart, c kidney, d liver, e lung, f lymph node, g muscle, and h spleen. X axis represents distances in bp. (PNG 14673 kb) [file 12864_2015_1938_MOESM19_ESM.png]

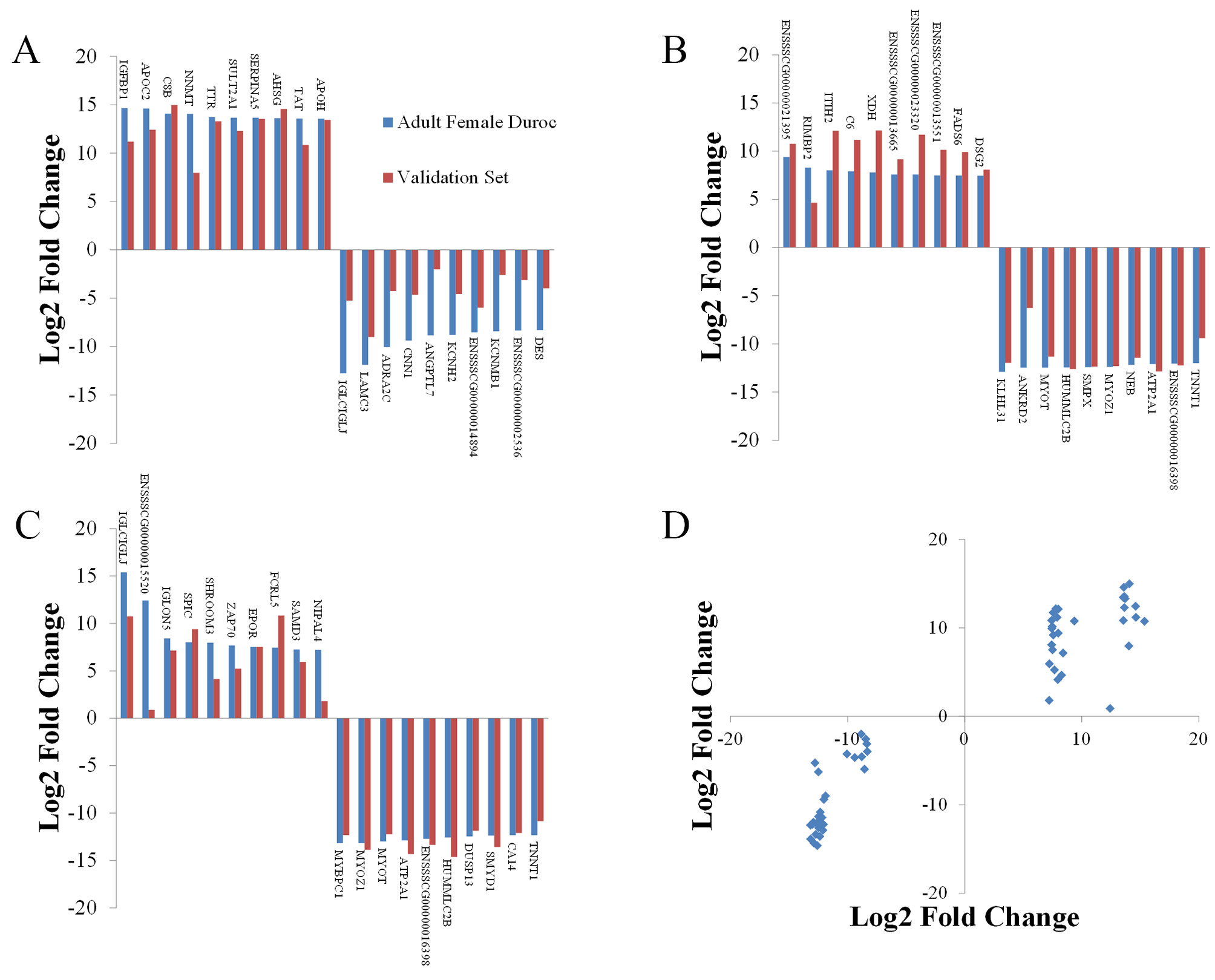

Supplement: Additional file 21: Figure S8. — Same directional gene expression changes across tissues in the adult female Duroc and validation datasets. Expression differences of the 10 most up- and downregulated genes between each tissue comparison from the adult female Duroc compared to the differences observed for the same genes in the validation dataset. a Log2 fold change differences in expression between liver and spleen, b liver and muscle, and c spleen and muscle. d Correlation between log2 fold changes in the adult female Duroc and validation dataset for all 3 tissue comparisons. (PNG 9535 kb) [file 12864_2015_1938_MOESM21_ESM.png]

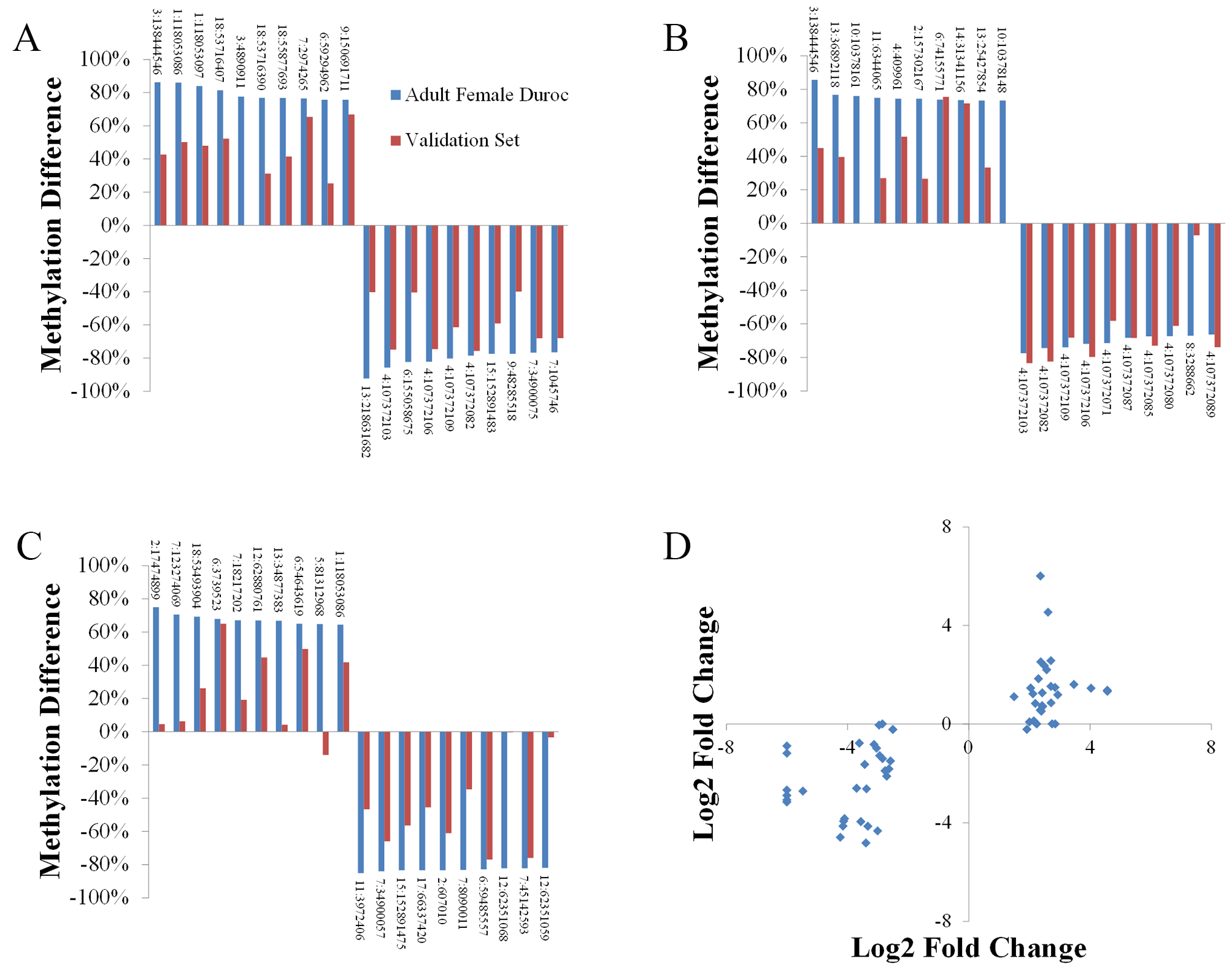

Supplement: Additional file 22: Figure S9. — Differences in CpG site methylation across tissues in the adult female Duroc and validation datasets. Methylation differences of the 10 most hyper and hypomethylated CpG sites between each tissue comparison from the adult female Duroc compared to the differences observed for the same sites in the validation dataset. a Methylation level differences at CpG sites between liver and muscle, b spleen and muscle, and c liver and spleen. d Correlation between log2 fold methylation changes in the adult female Duroc and validation dataset for all 3 tissue comparisons. (PNG 9329 kb) [file 12864_2015_1938_MOESM22_ESM.png]
